# *KRAS* mutations in uterine endometrium are associated with gravidity and parity

**DOI:** 10.1038/s41419-020-2559-0

**Published:** 2020-05-11

**Authors:** Satoshi Inoue, Emiko Yoshida, Yamato Fukui, Toshihide Ueno, Masahito Kawazu, Reina Takeyama, Masako Ikemura, Yutaka Osuga, Yasuhisa Terao, Yasushi Hirota, Hiroyuki Mano

**Affiliations:** 1 Division of Cellular Signaling, National Cancer Center Research Institute, Tokyo, 104-0045 Japan; 2 Department of Obstetrics and Gynecology, Juntendo University Faculty of Medicine, Tokyo, 113-8421 Japan; 3 Department of Obstetrics and Gynecology, The University of Tokyo, Tokyo, 113-0033 Japan; 4 Department of Pathology, Graduate School of Medicine, The University of Tokyo, Tokyo, 113-0033 Japan

**Keywords:** Molecular biology, Reproductive disorders

Recent genomic analyses have shown that, despite appearing histologically normal, many tissues bear somatic mutations. Somatic clones with genomic alterations can expand in such normal tissues due not only to aging but also to exposure to exogenous stresses or stimuli^1^. Benign disorders such as colitis can also stem from such genomic changes^2^. The uterus is the only organ experiencing menstruation, gestation and birth, and so the normal endometrium (NE) is exposed to unique stresses capable of inducing somatic genomic alterations. External physical/mechanical factors such as caesarean section can induce reactions that trigger genomic alterations in NE. Notably, recurrent mutations affecting *KRAS* and *PIK3CA* have been reported in NE^3–6^. Increased age, high body mass index and parity may be associated with a burden of mutations in NE^4,5^.

Adenomyosis is a common benign gynecological disorder in women of reproductive age that reduce the quality-of-life of affected women. Endometrium-like epithelial cells that ectopically proliferate in normal myometrium (NM). Mutation of *KRAS* and/or *PIK3CA* in NE is the earliest events in the molecular pathogenesis of adenomyosis^3^. It has been shown that parity, spontaneous abortion and smoking could be risk factors for adenomyosis^7^^,^^8^. In this study, we investigated whether particular clinical characteristics of individuals are associated with the presence of *KRAS, PIK3CA* and *PPP2R1A* alterations in NE.

In a previous study, we performed mutational analyses of NE from 56 individuals^3^. Here, we collected additional 42 uterine tissue samples, enabling us to examine *KRAS*, *PIK3CA* and *PPP2R1A* alterations in NE samples from a total of 98 women (Supplementary Tables S1 and S2). After enrichment of NE and NM by macro-dissection, genomic DNA from NE and NM (control) for each individual was subjected to targeted deep sequencings to assess mutations in the genomic hotspots *KRAS* p.G12/G13, *PIK3CA* p.H1047 and *PPP2R1A* p.P179/R182−R183 (Table 1, Supplementary Tables S3−S9), all of which have previously been reported as undergoing mutations in NE^3^. Consistent with recent publications^3–6^, we commonly observed mutations in *KRAS* p.G12/G13 (58/98 = 59.18%), *PIK3CA* p.H1047 (19/98 = 19.39%) and *PPP2R1A* p. P179/R182−R183 (15/98 = 15.31%) in the NE of individuals (Table 1, Supplementary Tables S10–S12). Most of clinical characteristics were not associated with any of these genetic alterations, and *PIK3CA* and *PPP2R1A* mutations were not linked to any specific clinical features (Table 1, Supplementary Tables S10–S12). However, mutations in *KRAS* p.G12/G13 were frequently detected in NE of individuals with vaginal delivery, gravidity and parity (Table 1, Supplelmentary Table S10). As *KRAS* p.G12/G13-mutated clones are known to be significantly expanded in NE of adenomyosis patients^3^, our observations raise the possibility that stresses during pregnancy and parity could induce mutations in *KRAS* in NE, providing a putative mechanism to explain why parity is a risk factor for adenomyosis. In contrast to vaginal delivery, no association between frequency of mutations in *KRAS* p.G12/G13 in NE and individuals with caesarean section were observed (Table 1, Supplementary Table S10), raising a possibility uterine contraction during vaginal delivery affect on the genomic alterations. Validation of our results in a larger patient cohort is obviously required, but if our findings are confirmed, they would support our hypothesis that gravidity and parity may drive adenomyosis pathogenesis, potentially explaining the increased frequency of *KRAS*-mutated clones in NE of these individuals.

**Table 1:** Relationship between individual characteristics and *KRAS* mutation status.

		Value (%)		
		*KRAS* wild type	*KRAS* mutated	
Characteristics		(*N* = 40)	(*N* = 58)	Statistics
Median age at operation (range), years		46 (35–87)	45 (33–65)	
Gravidity, *n* (%)	≥1	12/40 (30.00)	38/58 (65.52)	0.001
	0	28/40 (70.00)	20/58 (34.48)	
	1	5/40 (12.50)	16/58 (27.59)	
	2	5/40 (12.50)	11/58 (18.97)	
	≥3	2/40 (5.00)	11/58 (18.97)	
Parity, *n* (%)	≥1	11/40 (27.50)	33/58 (56.90)	0.007
	0	29/40 (72.50)	25/58 (43.10)	
	1	5/40 (12.50)	15/58 (25.86)	
	2	5/40 (12.50)	10/58 (17.24)	
	≥3	1/40 (2.50)	8/58 (13.79)	
Abortion or stillborn, *n* (%)	≥1	3/40 (7.50)	11/58 (18.97)	n.s.
	0	37/40 (92.50)	47/58 (81.03)	
	1	2/40 (5.00)	7/58 (12.07)	
	2	1/40 (2.50)	3/58 (5.17)	
	≥3	0/40 (0.00)	1/58 (1.72)	
Caesarean section, *n* (%)	≥1	6/40 (15.00)	5/58 (8.62)	n.s.
	0	34/40 (85.00)	53/58 (91.38)	
	1	4/40 (10.00)	2/58 (3.45)	
	2	2/40 (5.00)	1/58 (1.72)	
	≥3	0/40 (0.00)	2/58 (3.45)	
Vaginal delivery, *n* (%)	≥1	7/40 (17.50)	28/58 (48.28)	0.003
	0	33/40 (82.50)	30/58 (51.72)	
	1	4/40 (10.00)	13/58 (22.41)	
	2	3/40 (7.50)	10/58 (17.24)	
	≥3	0/40 (0.00)	5/58 (8.62)	
Smoking history, *n* (%)		8/40 (20.00)	5/58 (8.62)	n.s.

*n.s.* not significant assessed by Fisher’s exact test.

Our work may have significant clinical implications. Identification of *KRAS*-mutated clones in NE of parous women may allow clinicians to choose an alternative protocol that might prevent or mitigate adenomyosis development, perhaps maintaining a better quality-of life for these women. In addition, our genomic analyses of NE have yielded novel biological insights that could lead to the identification of new therapeutic strategies to eliminate *KRAS*-mutated clones in NE and prevent gravidity/parity-associated adenomyosis.

## Supplementary information

Supporting information
