# Supplementary material for: KRAS mutations in uterine endometrium are associated with gravidity and parity
Source: Cell Death Dis. 2020 May 11;11(5):347. doi: 10.1038/s41419-020-2559-0 (PMC7214428; doi:10.1038/s41419-020-2559-0)
Supplement: Supplementary file 1 — Supporting information [file 41419_2020_2559_MOESM1_ESM.docx]

**SUPPLEMENTARY INFORMATION**

**Supplementary Methods**

***Ethics and sample collection***

The institutional ethics committees of the University of Tokyo (Project Number G10035), the Juntendo University Faculty of Medicine (Project Number 2014176), and the National Cancer Center Research Institute (Project Number 2015−202) approved this project. Tissue samples from patients who gave written informed consent prior to their participation were obtained at the University of Tokyo Hospital and Juntendo University Hospital between May 2017 and February 2020. See also Table S2 for additional clinical details pertaining to these specimens.

***Targeted deep sequencing analyses of dissected endometrium and myometrium***

FFPE (Formalin-fixed paraffin-embedded) tissue samples were sectioned at 10 μm thickness and mounted on glass slides. NM and NE were macro-dissected under a microscope. Genomic DNA was extracted using the GeneRead DNA FFPE Kit (Qiagen). Primers are listed in Table S3. PCR products were subjected to library construction using the NEBNext Ultra DNA Library Prep Kit (New England BioLabs). Paired-end sequencing (150 bp) was conducted on an Illumina Miseq instrument using Reagent Kit V2 (300 cycles).

Most somatic genomic alterations, including *KRAS*, *PIK3CA* and *PPP2R1A* mutations, were detected only in NE and not in NM (Tables S4−9). As the VAFs (variant allele frequency) of the mutations in the dissected epithelial component of NE were low (Tables S4−6), we considered an alteration in a sample as a true mutation if its VAF was higher than the mean VAF + 3 standard deviations (SD) of highest position of each gene from the 98 NM samples (Table S7−9). The cutoffs for the somatic mutations are: 0.241% (mean: 0.034%, SD: 0.069%) for *KRAS* p.G12/G13, 0.146% (Mean: 0.026%, SD: 0.040%) for *PIK3CA* p.H1047, and 0.142% (Mean: 0.037%, SD: 0.035%) for *PPP2R1A* p. R179/R182−R183.

***Statistics***

Statistical analyses used for judging somatic mutations were described under the heading “Targeted deep sequencing analyses of dissected endometrium and myometrium” (Tables S7−9). Comparisons of the distribution of categorical variables in different groups were performed using Fisher’s exact test (Tables 1 and S10−12).

**Author contributions**: S.I., M.K. Y.O., Y.T., Y.H., and H.M. conceived the project and designed the study. E.Y., Y.F. Y.T., and Y.H. collected clinical samples. S.I. and R.T. performed NGS experiments. S.I. and T.U. performed NGS data analyses. M.I. performed histological analyses. S.I wrote and edited the manuscript with feedback from all authors.

**Data availability**: All data supporting the findings of this study are available in our Supplementary Information file or can be obtained from the corresponding author upon reasonable request.

**Code availability**: We employed publicly available algorithms for this study and did not use any custom code.

**Acknowledgement**: We thank members of Dr. Mano’s lab, particularly Ms. Takako Matsumoto, and National Cancer Center Research Institute Core Facility, particularly Ms. Yurika Shiotani and Mr. Uchiya, for their expert technical support. We are grateful to all the women who have generously donated their tissues to be used in our research studies; without them, this work would not have been possible. This study was supported in part by JSPS KAKENHI grants (#19K07708 to S.I, #19H03144 to Y.H, #17K07250 to T.U, #19H03796 to Y.O, and #18K09299 to Y.T); a grant for Project for “Whole Implementation to Support and Ensure the female life” (WISE) (JP #19gk0210021h0001 to Y.H.), Frontier Outstanding Research for Clinical Empowerment (FORCE) (JP#20gm4010010h0001 to Y.H.), a grant for Practical Research for Innovative Caner Control (JP#20ck0106539h0001 to S.I., U.T. and M.H.), a grant for Project for Cancer Research and Therapeutic Evolution (P-CREATE) (JP19cm0106502 to T.U. and M.K.) from the Japanese Agency for Medical Research and Development; and a grant for The Kurata Grants from The Hitachi Global Foundation and The Foundation for Promotion of Cancer Research in Japan (to S.I.).

**Supplementary Tables (Table S1-12)**

| **Table S1. Clinical characteristics of individual in the normal uterine endometrium cohort.** | |
| --- | --- |
| Characteristics | Value |
| Median age at operation (range) –yr | 45 (33-87) |
|  |  |
| History |  |
| Gravidity –no. (%) | 50 (51.02) |
| Parity –no. (%) | 44 (44.90) |
| Abortion or Stillborn –no. (%) | 14 (14.29) |
| Caesarean section –no. (%) | 11 (11.22) |
| Vaginal delivery –no. (%) | 35 (35.71) |
| Smoking history –no. (%) | 13 (13.27) |

| **Table S2. Clinical characteristics of individuals in the present study.** | | | | | | | | | |  |  |
| --- | --- | --- | --- | --- | --- | --- | --- | --- | --- | --- | --- |
| ID | Age | CS | DC | VD | AS | G | P | SH | *KRAS* | *PIK3* | *PPP* |
| 01 | 43 | 0 | 0 | 2 | 0 | 2 | 2 | 0 | 1 | 0 | 0 |
| 02 | 45 | 0 | 0 | 1 | 0 | 1 | 1 | 0 | 1 | 0 | 0 |
| 03 | 43 | 0 | 0 | 0 | 0 | 0 | 0 | 0 | 0 | 0 | 0 |
| 04 | 47 | 0 | 0 | 3 | 0 | 3 | 3 | 0 | 1 | 0 | 0 |
| 05 | 45 | 0 | 0 | 0 | 0 | 0 | 0 | 0 | 1 | 0 | 0 |
| 06 | 45 | 0 | 0 | 2 | 0 | 2 | 2 | 0 | 1 | 0 | 0 |
| 07 | 39 | 0 | 0 | 0 | 0 | 0 | 0 | 0 | 1 | 0 | 0 |
| 08 | 43 | 0 | 0 | 0 | 0 | 0 | 0 | 0 | 1 | 1 | 0 |
| 09 | 43 | 0 | 0 | 1 | 0 | 1 | 1 | 0 | 1 | 0 | 0 |
| 10 | 40 | 1 | 0 | 0 | 0 | 1 | 1 | 0 | 1 | 0 | 1 |
| 11 | 52 | 0 | 0 | 0 | 0 | 0 | 0 | 0 | 0 | 0 | 0 |
| 12 | 44 | 0 | 0 | 0 | 0 | 0 | 0 | 1 | 0 | 0 | 0 |
| 13 | 41 | 0 | 0 | 3 | 0 | 3 | 3 | 1 | 1 | 0 | 0 |
| 14 | 45 | 0 | 0 | 0 | 0 | 0 | 0 | 0 | 0 | 0 | 0 |
| 15 | 44 | 0 | 0 | 2 | 0 | 2 | 2 | 1 | 1 | 0 | 0 |
| 16 | 48 | 0 | 0 | 0 | 0 | 0 | 0 | 0 | 0 | 0 | 0 |
| 17 | 42 | 0 | 0 | 0 | 0 | 0 | 0 | 0 | 0 | 0 | 0 |
| 18 | 47 | 0 | 3 | 3 | 3 | 6 | 3 | 0 | 1 | 0 | 0 |
| 19 | 40 | 0 | 0 | 0 | 0 | 0 | 0 | 1 | 0 | 0 | 0 |
| 20 | 44 | 0 | 1 | 0 | 1 | 1 | 0 | 1 | 1 | 0 | 0 |
| 21 | 40 | 0 | 1 | 1 | 1 | 2 | 1 | 0 | 1 | 1 | 0 |
| 22 | 39 | 0 | 1 | 0 | 1 | 1 | 0 | 0 | 1 | 0 | 0 |
| 23 | 45 | 0 | 0 | 1 | 0 | 1 | 1 | 0 | 1 | 0 | 0 |
| 24 | 41 | 0 | 1 | 0 | 2 | 2 | 0 | 0 | 1 | 0 | 0 |
| 25 | 47 | 0 | 0 | 2 | 0 | 2 | 2 | 0 | 1 | 0 | 0 |
| 26 | 40 | 0 | 1 | 0 | 1 | 1 | 0 | 1 | 0 | 0 | 0 |
| 27 | 44 | 2 | 2 | 0 | 2 | 4 | 2 | 1 | 0 | 0 | 0 |
| 28 | 46 | 0 | 0 | 0 | 0 | 0 | 0 | 0 | 1 | 0 | 0 |
| 29 | 47 | 0 | 0 | 0 | 0 | 0 | 0 | 0 | 1 | 0 | 0 |
| 30 | 49 | 0 | 0 | 0 | 0 | 0 | 0 | 0 | 0 | 0 | 0 |
| 31 | 44 | 0 | 0 | 0 | 0 | 0 | 0 | 0 | 0 | 0 | 0 |
| 32 | 43 | 0 | 0 | 2 | 0 | 2 | 2 | 0 | 1 | 0 | 0 |
| 33 | 46 | 0 | 0 | 0 | 0 | 0 | 0 | 0 | 0 | 0 | 0 |
| 34 | 42 | 0 | 0 | 2 | 0 | 2 | 2 | 0 | 0 | 0 | 0 |
| 35 | 50 | 0 | 0 | 1 | 0 | 1 | 1 | 0 | 0 | 0 | 0 |
| 36 | 44 | 2 | 0 | 0 | 0 | 2 | 2 | 0 | 0 | 0 | 0 |
| 37 | 47 | 0 | 0 | 0 | 0 | 0 | 0 | 1 | 1 | 1 | 0 |
| 38 | 46 | 1 | 0 | 0 | 0 | 1 | 1 | 0 | 1 | 0 | 0 |
| 39 | 49 | 1 | 0 | 2 | 0 | 3 | 3 | 0 | 0 | 1 | 0 |
| 40 | 44 | 0 | 0 | 0 | 0 | 0 | 0 | 0 | 1 | 1 | 1 |
| 41 | 45 | 0 | 0 | 1 | 0 | 1 | 1 | 0 | 0 | 0 | 0 |
| 42 | 52 | 0 | 0 | 0 | 0 | 0 | 0 | 0 | 0 | 1 | 1 |
| 43 | 46 | 1 | 0 | 1 | 0 | 2 | 2 | 0 | 0 | 0 | 1 |
| 44 | 45 | 0 | 0 | 0 | 0 | 0 | 0 | 1 | 0 | 0 | 1 |
| 45 | 51 | 0 | 0 | 1 | 0 | 1 | 1 | 0 | 1 | 0 | 1 |
| 46 | 46 | 0 | 0 | 0 | 0 | 0 | 0 | 1 | 0 | 0 | 0 |
| 47 | 47 | 0 | 0 | 0 | 0 | 0 | 0 | 0 | 1 | 0 | 1 |
| 48 | 38 | 0 | 0 | 2 | 0 | 2 | 2 | 0 | 1 | 1 | 0 |
| 49 | 43 | 0 | 0 | 0 | 0 | 0 | 0 | 0 | 0 | 0 | 0 |
| 50 | 43 | 0 | 0 | 2 | 0 | 2 | 2 | 0 | 1 | 0 | 1 |
| 51 | 47 | 0 | 0 | 1 | 0 | 1 | 1 | 0 | 0 | 1 | 1 |
| 52 | 49 | 0 | 0 | 0 | 0 | 0 | 0 | 0 | 0 | 1 | 0 |
| 53 | 42 | 0 | 0 | 0 | 0 | 0 | 0 | 0 | 0 | 0 | 0 |
| 54 | 47 | 0 | 0 | 2 | 0 | 2 | 2 | 0 | 0 | 0 | 0 |
| 55 | 46 | 0 | 0 | 0 | 0 | 0 | 0 | 0 | 0 | 0 | 0 |
| 56 | 45 | 0 | 0 | 0 | 0 | 0 | 0 | 0 | 0 | 0 | 0 |
| 57 | 51 | 0 | 0 | 0 | 0 | 0 | 0 | 0 | 0 | 1 | 0 |
| 58 | 48 | 0 | 0 | 0 | 0 | 0 | 0 | 0 | 0 | 1 | 1 |
| 59 | 41 | 0 | 0 | 0 | 0 | 0 | 0 | 0 | 1 | 0 | 0 |
| 60 | 44 | 3 | 0 | 0 | 0 | 3 | 3 | 0 | 1 | 0 | 0 |
| 61 | 43 | 0 | 0 | 3 | 0 | 3 | 3 | 0 | 1 | 0 | 0 |
| 62 | 38 | 0 | 2 | 1 | 2 | 3 | 1 | 0 | 1 | 0 | 0 |
| 63 | 46 | 0 | 0 | 1 | 0 | 1 | 1 | 0 | 1 | 0 | 0 |
| 64 | 48 | 0 | 0 | 1 | 0 | 1 | 1 | 0 | 1 | 0 | 0 |
| 65 | 45 | 0 | 0 | 0 | 0 | 0 | 0 | 0 | 1 | 0 | 0 |
| 66 | 41 | 0 | 0 | 0 | 0 | 0 | 0 | 0 | 1 | 1 | 0 |
| 67 | 43 | 1 | 0 | 0 | 1 | 2 | 1 | 0 | 0 | 0 | 0 |
| 68 | 43 | 0 | 0 | 2 | 0 | 2 | 2 | 1 | 1 | 0 | 0 |
| 69 | 50 | 0 | 0 | 0 | 0 | 0 | 0 | 0 | 1 | 0 | 0 |
| 70 | 42 | 0 | 0 | 0 | 0 | 1 | 1 | 0 | 1 | 0 | 0 |
| 71 | 51 | 3 | 0 | 0 | 0 | 3 | 3 | 0 | 1 | 1 | 0 |
| 72 | 50 | 0 | 0 | 0 | 0 | 0 | 0 | 0 | 1 | 0 | 1 |
| 73 | 37 | 0 | 0 | 0 | 0 | 0 | 0 | 0 | 1 | 0 | 0 |
| 74 | 51 | 1 | 0 | 0 | 0 | 1 | 1 | 0 | 0 | 0 | 0 |
| 75 | 35 | 0 | 0 | 0 | 0 | 0 | 0 | 0 | 0 | 1 | 0 |
| 76 | 46 | 0 | 0 | 0 | 0 | 0 | 0 | 0 | 1 | 1 | 0 |
| 77 | 42 | 0 | 0 | 2 | 0 | 2 | 2 | 0 | 1 | 0 | 0 |
| 78 | 65 | 2 | 0 | 1 | 0 | 3 | 3 | 0 | 1 | 0 | 0 |
| 79 | 48 | 0 | 0 | 0 | 0 | 0 | 0 | 0 | 1 | 1 | 0 |
| 80 | 42 | 0 | 0 | 0 | 0 | 0 | 0 | 0 | 1 | 0 | 0 |
| 81 | 42 | 0 | 0 | 0 | 0 | 0 | 0 | 0 | 1 | 0 | 0 |
| 82 | 49 | 0 | 0 | 0 | 0 | 0 | 0 | 0 | 0 | 0 | 1 |
| 83 | 33 | 0 | 1 | 1 | 1 | 3 | 1 | 0 | 1 | 1 | 1 |
| 84 | 41 | 0 | 0 | 0 | 0 | 0 | 0 | 0 | 0 | 0 | 0 |
| 85 | 44 | 0 | 0 | 0 | 0 | 0 | 0 | 0 | 1 | 0 | 0 |
| 86 | 48 | 0 | 2 | 2 | 2 | 4 | 2 | 0 | 1 | 0 | 0 |
| 87 | 50 | 0 | 0 | 1 | 0 | 1 | 1 | 0 | 1 | 1 | 0 |
| 88 | 46 | 0 | 0 | 0 | 0 | 0 | 0 | 0 | 1 | 0 | 0 |
| 89 | 52 | 0 | 0 | 0 | 1 | 1 | 0 | 0 | 1 | 0 | 0 |
| 90 | 55 | 0 | 0 | 0 | 0 | 0 | 0 | 0 | 0 | 0 | 1 |
| 91 | 63 | 0 | 1 | 3 | 1 | 4 | 3 | 0 | 1 | 0 | 0 |
| 92 | 49 | 0 | 0 | 0 | 0 | 0 | 0 | 1 | 0 | 0 | 0 |
| 93 | 55 | 0 | 0 | 0 | 0 | 0 | 0 | 0 | 0 | 0 | 0 |
| 94 | 58 | 0 | 1 | 0 | 1 | 1 | 0 | 0 | 1 | 1 | 0 |
| 95 | 87 | 0 | 0 | 0 | 0 | 0 | 0 | 0 | 0 | 0 | 0 |
| 96 | 53 | 0 | 0 | 1 | 0 | 1 | 1 | 0 | 1 | 0 | 0 |
| 97 | 45 | 0 | 0 | 1 | 0 | 1 | 1 | 0 | 1 | 0 | 0 |
| 98 | 47 | 0 | 0 | 0 | 0 | 0 | 0 | 1 | 0 | 0 | 1 |
| CS: Caesarean section; DC: Dilatation and curettage; VD: Vaginal delivery | | | | | | | | | |  |  |
| AS: Abortion or stillborn; G: Gravidity, P: Parity, SH: Smoking history | | | | | | | | |  |  |  |
| *KRAS*: Mutations in *KRAS* p.G12/G13; *PIK*: Mutations in *PIK3CA* p.H1047  *PPP*: Mutations in PPP2R1A p.P179/R182-183 | | | | | | | | | |  |  |

| **Table S4. Summary of targeted deep sequencing read information for *KRAS* mutation analyses.** | | | | | | | |
| --- | --- | --- | --- | --- | --- | --- | --- |
| ID | Chr | Position | Ref>Mut | Total reads | Mutation reads | VAF (%) | Somatic mut^1^ |
| 07NE | chr12 | 25245351 | C>A | 15406 | 1040 | 6.751 | YES |
| 62NE | chr12 | 25245351 | C>A | 16934 | 1142 | 6.744 | YES |
| 65NE | chr12 | 25245351 | C>A | 16665 | 1085 | 6.511 | YES |
| 24NE | chr12 | 25245351 | C>A | 14449 | 875 | 6.056 | YES |
| 23NE | chr12 | 25245351 | C>A | 31075 | 1837 | 5.912 | YES |
| 96NE | chr12 | 25245351 | C>A | 18167 | 956 | 5.262 | YES |
| 68NE | chr12 | 25245350 | C>G | 5515 | 262 | 4.751 | YES |
| 70NE | chr12 | 25245350 | C>A | 3198 | 136 | 4.253 | YES |
| 06NE | chr12 | 25245350 | C>T | 11212 | 441 | 3.933 | YES |
| 85NE | chr12 | 25245350 | C>T | 8984 | 352 | 3.918 | YES |
| 61NE | chr12 | 25245350 | C>T | 10844 | 404 | 3.726 | YES |
| 10NE | chr12 | 25245350 | C>T | 23053 | 841 | 3.648 | YES |
| 86NE | chr12 | 25245351 | C>A | 7875 | 285 | 3.619 | YES |
| 79NE | chr12 | 25245350 | C>A | 9324 | 324 | 3.475 | YES |
| 69NE | chr12 | 25245350 | C>T | 6307 | 193 | 3.060 | YES |
| 66NE | chr12 | 25245351 | C>A | 4308 | 124 | 2.878 | YES |
| 77NE | chr12 | 25245347 | C>T | 14046 | 351 | 2.499 | YES |
| 29NE | chr12 | 25245350 | C>A | 2089 | 49 | 2.346 | YES |
| 97NE | chr12 | 25245350 | C>T | 11843 | 214 | 1.807 | YES |
| 78NE | chr12 | 25245350 | C>A | 3319 | 57 | 1.717 | YES |
| 13NE | chr12 | 25245347 | C>A | 3774 | 64 | 1.696 | YES |
| 94NE | chr12 | 25245350 | C>T | 18456 | 297 | 1.609 | YES |
| 15NE | chr12 | 25245347 | C>A | 3418 | 55 | 1.609 | YES |
| 20NE | chr12 | 25245347 | C>A | 4173 | 67 | 1.606 | YES |
| 22NE | chr12 | 25245347 | C>A | 4316 | 68 | 1.576 | YES |
| 32NE | chr12 | 25245350 | C>T | 4264 | 65 | 1.524 | YES |
| 05NE | chr12 | 25245350 | C>T | 14854 | 222 | 1.495 | YES |
| 18NE | chr12 | 25245347 | C>A | 4033 | 60 | 1.488 | YES |
| 60NE | chr12 | 25245350 | C>T | 16356 | 243 | 1.486 | YES |
| 21NE | chr12 | 25245347 | C>A | 3791 | 56 | 1.477 | YES |
| 81NE | chr12 | 25245351 | C>A | 10030 | 145 | 1.446 | YES |
| 80NE | chr12 | 25245351 | C>A | 14727 | 204 | 1.385 | YES |
| 91NE | chr12 | 25245350 | C>T | 19909 | 269 | 1.351 | YES |
| 76NE | chr12 | 25245350 | C>A | 7113 | 95 | 1.336 | YES |
| 25NE | chr12 | 25245350 | C>T | 13576 | 180 | 1.326 | YES |
| 50NE | chr12 | 25245350 | C>T | 31556 | 388 | 1.230 | YES |
| 64NE | chr12 | 25245350 | C>T | 34110 | 412 | 1.208 | YES |
| 87NE | chr12 | 25245350 | C>T | 11588 | 134 | 1.156 | YES |
| 63NE | chr12 | 25245350 | C>T | 13577 | 146 | 1.075 | YES |
| 02NE | chr12 | 25245350 | C>T | 16604 | 172 | 1.036 | YES |
| 83NE | chr12 | 25245350 | C>A | 5190 | 52 | 1.002 | YES |
| 08NE | chr12 | 25245350 | C>T | 15156 | 151 | 0.996 | YES |
| 37NE | chr12 | 25245350 | C>A | 12034 | 117 | 0.972 | YES |
| 04NE | chr12 | 25245350 | C>T | 15774 | 138 | 0.875 | YES |
| 01NE | chr12 | 25245350 | C>T | 13275 | 115 | 0.866 | YES |
| 89NE | chr12 | 25245350 | C>T | 17539 | 146 | 0.832 | YES |
| 73NE | chr12 | 25245350 | C>T | 9256 | 75 | 0.810 | YES |
| 22NE | chr12 | 25245351 | C>A | 4467 | 35 | 0.784 | YES |
| 72NE | chr12 | 25245351 | C>A | 7684 | 57 | 0.742 | YES |
| 38NE | chr12 | 25245350 | C>G | 9618 | 69 | 0.717 | YES |
| 59NE | chr12 | 25245351 | C>A | 26530 | 187 | 0.705 | YES |
| 45NE | chr12 | 25245350 | C>A | 16763 | 112 | 0.668 | YES |
| 71NE | chr12 | 25245350 | C>T | 5352 | 35 | 0.654 | YES |
| 13NE | chr12 | 25245351 | C>A | 3876 | 23 | 0.593 | YES |
| 69NE | chr12 | 25245347 | C>T | 6265 | 34 | 0.543 | YES |
| 13NE | chr12 | 25245348 | C>A | 3816 | 17 | 0.445 | YES |
| 15NE | chr12 | 25245348 | C>A | 3453 | 15 | 0.434 | YES |
| 20NE | chr12 | 25245348 | C>A | 4236 | 14 | 0.331 | YES |
| 22NE | chr12 | 25245348 | C>A | 4412 | 14 | 0.317 | YES |
| 70NE | chr12 | 25245351 | C>A | 3184 | 10 | 0.314 | YES |
| 09NE | chr12 | 25245350 | C>T | 18619 | 58 | 0.312 | YES |
| 18NE | chr12 | 25245348 | C>A | 4100 | 12 | 0.293 | YES |
| 48NE | chr12 | 25245348 | C>T | 14661 | 40 | 0.273 | YES |
| 28NE | chr12 | 25245350 | C>A | 1118 | 3 | 0.268 | YES |
| 88NE | chr12 | 25245350 | C>T | 31325 | 83 | 0.265 | YES |
| 47NE | chr12 | 25245350 | C>T | 17509 | 45 | 0.257 | YES |
| 40NE | chr12 | 25245351 | C>A | 16867 | 43 | 0.255 | YES |
| 84NE | chr12 | 25245350 | C>T | 5130 | 12 | 0.234 | NO |
| 75NE | chr12 | 25245351 | C>A | 9542 | 22 | 0.231 | NO |
| 75NE | chr12 | 25245350 | C>G | 9591 | 22 | 0.229 | NO |
| 45NE | chr12 | 25245348 | C>A | 16692 | 38 | 0.228 | NO |
| 31NE | chr12 | 25245350 | C>A | 6457 | 14 | 0.217 | NO |
| 21NE | chr12 | 25245348 | C>A | 3884 | 8 | 0.206 | NO |
| 30NE | chr12 | 25245350 | C>A | 7322 | 15 | 0.205 | NO |
| 82NE | chr12 | 25245350 | C>A | 12954 | 24 | 0.185 | NO |
| 33NE | chr12 | 25245350 | C>A | 4354 | 8 | 0.184 | NO |
| 28NE | chr12 | 25245348 | C>A | 1124 | 2 | 0.178 | NO |
| 03NE | chr12 | 25245350 | C>T | 17227 | 25 | 0.145 | NO |
| 24NE | chr12 | 25245350 | C>A | 14351 | 20 | 0.139 | NO |
| 90NE | chr12 | 25245350 | C>T | 15077 | 20 | 0.133 | NO |
| 44NE | chr12 | 25245347 | C>T | 10082 | 13 | 0.129 | NO |
| 27NE | chr12 | 25245350 | C>A | 12942 | 16 | 0.124 | NO |
| 26NE | chr12 | 25245350 | C>A | 21251 | 26 | 0.122 | NO |
| 13NE | chr12 | 25245349 | A>C | 3795 | 4 | 0.105 | NO |
| 67NE | chr12 | 25245350 | C>A | 2940 | 3 | 0.102 | NO |
| 39NE | chr12 | 25245350 | C>G | 14846 | 14 | 0.094 | NO |
| 22NE | chr12 | 25245349 | A>C | 4366 | 4 | 0.092 | NO |
| 56NE | chr12 | 25245350 | C>A | 11284 | 10 | 0.089 | NO |
| 43NE | chr12 | 25245347 | C>T | 13228 | 10 | 0.076 | NO |
| 20NE | chr12 | 25245349 | A>C | 4191 | 3 | 0.072 | NO |
| 76NE | chr12 | 25245347 | C>T | 7080 | 5 | 0.071 | NO |
| 66NE | chr12 | 25245350 | C>A | 4281 | 3 | 0.070 | NO |
| 67NE | chr12 | 25245348 | C>A | 2968 | 2 | 0.067 | NO |
| 52NE | chr12 | 25245348 | C>T | 1527 | 1 | 0.065 | NO |
| 73NE | chr12 | 25245351 | C>T | 9180 | 6 | 0.065 | NO |
| 52NE | chr12 | 25245349 | A>G | 1537 | 1 | 0.065 | NO |
| 11NE | chr12 | 25245351 | C>A | 3818 | 2 | 0.052 | NO |
| 74NE | chr12 | 25245351 | C>T | 22650 | 11 | 0.049 | NO |
| 74NE | chr12 | 25245350 | C>T | 22880 | 11 | 0.048 | NO |
| 29NE | chr12 | 25245349 | A>T | 2105 | 1 | 0.048 | NO |
| 20NE | chr12 | 25245351 | C>A | 4290 | 2 | 0.047 | NO |
| 33NE | chr12 | 25245349 | A>C | 4388 | 2 | 0.046 | NO |
| 59NE | chr12 | 25245350 | C>T | 26862 | 11 | 0.041 | NO |
| 84NE | chr12 | 25245349 | A>C | 5083 | 2 | 0.039 | NO |
| 63NE | chr12 | 25245351 | C>A | 13496 | 5 | 0.037 | NO |
| 14NE | chr12 | 25245347 | C>A | 25819 | 9 | 0.035 | NO |
| 67NE | chr12 | 25245351 | C>A | 2963 | 1 | 0.034 | NO |
| 85NE | chr12 | 25245351 | C>T | 8908 | 3 | 0.034 | NO |
| 88NE | chr12 | 25245351 | C>A | 31091 | 10 | 0.032 | NO |
| 70NE | chr12 | 25245349 | AC>A | 3197 | 1 | 0.031 | NO |
| 12NE | chr12 | 25245350 | C>T | 6490 | 2 | 0.031 | NO |
| 60NE | chr12 | 25245348 | C>T | 16331 | 5 | 0.031 | NO |
| 95NE | chr12 | 25245351 | C>T | 6615 | 2 | 0.030 | NO |
| 78NE | chr12 | 25245351 | C>A | 3319 | 1 | 0.030 | NO |
| 78NE | chr12 | 25245349 | A>G | 3321 | 1 | 0.030 | NO |
| 92NE | chr12 | 25245348 | C>T | 13376 | 4 | 0.030 | NO |
| 45NE | chr12 | 25245349 | A>T | 16795 | 5 | 0.030 | NO |
| 95NE | chr12 | 25245350 | C>T | 6728 | 2 | 0.030 | NO |
| 15NE | chr12 | 25245349 | A>C | 3438 | 1 | 0.029 | NO |
| 46NE | chr12 | 25245348 | C>T | 13886 | 4 | 0.029 | NO |
| 36NE | chr12 | 25245348 | C>T | 24813 | 7 | 0.028 | NO |
| 30NE | chr12 | 25245351 | C>A | 7355 | 2 | 0.027 | NO |
| 30NE | chr12 | 25245348 | C>T | 7366 | 2 | 0.027 | NO |
| 96NE | chr12 | 25245348 | C>T | 18453 | 5 | 0.027 | NO |
| 11NE | chr12 | 25245348 | C>T | 3832 | 1 | 0.026 | NO |
| 21NE | chr12 | 25245349 | A>C | 3834 | 1 | 0.026 | NO |
| 41NE | chr12 | 25245348 | C>T | 11509 | 3 | 0.026 | NO |
| 11NE | chr12 | 25245349 | A>G | 3839 | 1 | 0.026 | NO |
| 11NE | chr12 | 25245350 | C>A | 3843 | 1 | 0.026 | NO |
| 34NE | chr12 | 25245351 | C>T | 27248 | 7 | 0.026 | NO |
| 21NE | chr12 | 25245351 | C>T | 3921 | 1 | 0.026 | NO |
| 97NE | chr12 | 25245347 | C>T | 11808 | 3 | 0.025 | NO |
| 86NE | chr12 | 25245348 | C>T | 7933 | 2 | 0.025 | NO |
| 18NE | chr12 | 25245349 | A>C | 4064 | 1 | 0.025 | NO |
| 36NE | chr12 | 25245351 | C>T | 24808 | 6 | 0.024 | NO |
| 82NE | chr12 | 25245349 | A>T | 12948 | 3 | 0.023 | NO |
| 66NE | chr12 | 25245349 | A>T | 4339 | 1 | 0.023 | NO |
| 33NE | chr12 | 25245351 | C>A | 4371 | 1 | 0.023 | NO |
| 33NE | chr12 | 25245347 | C>T | 4376 | 1 | 0.023 | NO |
| 22NE | chr12 | 25245350 | C>A | 4396 | 1 | 0.023 | NO |
| 43NE | chr12 | 25245348 | C>T | 13294 | 3 | 0.023 | NO |
| 94NE | chr12 | 25245351 | C>T | 18112 | 4 | 0.022 | NO |
| 96NE | chr12 | 25245349 | A>C | 18287 | 4 | 0.022 | NO |
| 94NE | chr12 | 25245349 | A>T | 18315 | 4 | 0.022 | NO |
| 10NE | chr12 | 25245351 | C>A | 22907 | 5 | 0.022 | NO |
| 73NE | chr12 | 25245347 | C>A | 9181 | 2 | 0.022 | NO |
| 16NE | chr12 | 25245351 | C>T | 46343 | 10 | 0.022 | NO |
| 46NE | chr12 | 25245349 | A>T | 13935 | 3 | 0.022 | NO |
| 38NE | chr12 | 25245351 | C>A | 9599 | 2 | 0.021 | NO |
| 38NE | chr12 | 25245349 | A>T | 9608 | 2 | 0.021 | NO |
| 48NE | chr12 | 25245349 | A>G | 14648 | 3 | 0.020 | NO |
| 80NE | chr12 | 25245347 | C>A | 14702 | 3 | 0.020 | NO |
| 91NE | chr12 | 25245349 | A>C | 19766 | 4 | 0.020 | NO |
| 05NE | chr12 | 25245349 | A>T | 14834 | 3 | 0.020 | NO |
| 39NE | chr12 | 25245349 | A>G | 14836 | 3 | 0.020 | NO |
| 90NE | chr12 | 25245349 | A>C | 14935 | 3 | 0.020 | NO |
| 08NE | chr12 | 25245351 | C>A | 15064 | 3 | 0.020 | NO |
| 84NE | chr12 | 25245351 | C>T | 5037 | 1 | 0.020 | NO |
| 44NE | chr12 | 25245348 | C>T | 10117 | 2 | 0.020 | NO |
| 83NE | chr12 | 25245351 | C>T | 5214 | 1 | 0.019 | NO |
| 88NE | chr12 | 25245349 | A>G | 31342 | 6 | 0.019 | NO |
| 83NE | chr12 | 25245349 | A>T | 5237 | 1 | 0.019 | NO |
| 71NE | chr12 | 25245349 | A>G | 5353 | 1 | 0.019 | NO |
| 26NE | chr12 | 25245348 | C>T | 21441 | 4 | 0.019 | NO |
| 61NE | chr12 | 25245351 | C>A | 10777 | 2 | 0.019 | NO |
| 68NE | chr12 | 25245348 | C>T | 5505 | 1 | 0.018 | NO |
| 68NE | chr12 | 25245349 | A>G | 5524 | 1 | 0.018 | NO |
| 02NE | chr12 | 25245348 | C>A | 16587 | 3 | 0.018 | NO |
| 35NE | chr12 | 25245349 | A>T | 11081 | 2 | 0.018 | NO |
| 65NE | chr12 | 25245347 | C>T | 16657 | 3 | 0.018 | NO |
| 40NE | chr12 | 25245347 | C>T | 16800 | 3 | 0.018 | NO |
| 06NE | chr12 | 25245348 | C>T | 11201 | 2 | 0.018 | NO |
| 06NE | chr12 | 25245349 | A>T | 11205 | 2 | 0.018 | NO |
| 64NE | chr12 | 25245351 | C>A | 33870 | 6 | 0.018 | NO |
| 62NE | chr12 | 25245349 | A>G | 17062 | 3 | 0.018 | NO |
| 03NE | chr12 | 25245351 | C>A | 17115 | 3 | 0.018 | NO |
| 10NE | chr12 | 25245349 | A>T | 23082 | 4 | 0.017 | NO |
| 87NE | chr12 | 25245348 | C>T | 11566 | 2 | 0.017 | NO |
| 89NE | chr12 | 25245349 | A>C | 17403 | 3 | 0.017 | NO |
| 97NE | chr12 | 25245351 | C>A | 11623 | 2 | 0.017 | NO |
| 19NE | chr12 | 25245348 | C>T | 35043 | 6 | 0.017 | NO |
| 19NE | chr12 | 25245349 | A>T | 35136 | 6 | 0.017 | NO |
| 93NE | chr12 | 25245351 | C>T | 11737 | 2 | 0.017 | NO |
| 97NE | chr12 | 25245349 | A>C | 11745 | 2 | 0.017 | NO |
| 49NE | chr12 | 25245349 | A>G | 11832 | 2 | 0.017 | NO |
| 93NE | chr12 | 25245349 | A>C | 11852 | 2 | 0.017 | NO |
| 37NE | chr12 | 25245349 | A>T | 12015 | 2 | 0.017 | NO |
| 96NE | chr12 | 25245350 | C>T | 18462 | 3 | 0.016 | NO |
| 09NE | chr12 | 25245351 | C>A | 18513 | 3 | 0.016 | NO |
| 36NE | chr12 | 25245349 | A>T | 24824 | 4 | 0.016 | NO |
| 23NE | chr12 | 25245347 | C>T | 31055 | 5 | 0.016 | NO |
| 69NE | chr12 | 25245349 | A>T | 6307 | 1 | 0.016 | NO |
| 98NE | chr12 | 25245349 | A>G | 44243 | 7 | 0.016 | NO |
| 27NE | chr12 | 25245351 | C>T | 13010 | 2 | 0.015 | NO |
| 27NE | chr12 | 25245348 | C>T | 13034 | 2 | 0.015 | NO |
| 27NE | chr12 | 25245349 | A>C | 13082 | 2 | 0.015 | NO |
| 31NE | chr12 | 25245349 | A>C | 6543 | 1 | 0.015 | NO |
| 91NE | chr12 | 25245347 | C>T | 19819 | 3 | 0.015 | NO |
| 16NE | chr12 | 25245349 | A>T | 46354 | 7 | 0.015 | NO |
| 92NE | chr12 | 25245349 | A>C | 13291 | 2 | 0.015 | NO |
| 95NE | chr12 | 25245349 | AC>A | 6694 | 1 | 0.015 | NO |
| 95NE | chr12 | 25245348 | C>G | 6725 | 1 | 0.015 | NO |
| 63NE | chr12 | 25245347 | C>A | 13457 | 2 | 0.015 | NO |
| 17NE | chr12 | 25245349 | A>G | 53960 | 8 | 0.015 | NO |
| 17NE | chr12 | 25245348 | C>T | 54008 | 8 | 0.015 | NO |
| 63NE | chr12 | 25245349 | A>C | 13557 | 2 | 0.015 | NO |
| 34NE | chr12 | 25245348 | C>T | 27254 | 4 | 0.015 | NO |
| 25NE | chr12 | 25245349 | A>C | 13696 | 2 | 0.015 | NO |
| 55NE | chr12 | 25245348 | C>T | 20833 | 3 | 0.014 | NO |
| 46NE | chr12 | 25245350 | C>A | 13899 | 2 | 0.014 | NO |
| 19NE | chr12 | 25245351 | C>A | 34936 | 5 | 0.014 | NO |
| 77NE | chr12 | 25245349 | A>T | 14136 | 2 | 0.014 | NO |
| 76NE | chr12 | 25245351 | C>T | 7085 | 1 | 0.014 | NO |
| 76NE | chr12 | 25245348 | C>T | 7109 | 1 | 0.014 | NO |
| 26NE | chr12 | 25245351 | C>T | 21408 | 3 | 0.014 | NO |
| 24NE | chr12 | 25245349 | A>C | 14533 | 2 | 0.014 | NO |
| 58NE | chr12 | 25245348 | C>T | 29277 | 4 | 0.014 | NO |
| 39NE | chr12 | 25245347 | C>A | 14759 | 2 | 0.014 | NO |
| 30NE | chr12 | 25245349 | A>G | 7383 | 1 | 0.014 | NO |
| 58NE | chr12 | 25245349 | A>T | 29576 | 4 | 0.014 | NO |
| 39NE | chr12 | 25245348 | C>T | 14795 | 2 | 0.014 | NO |
| 39NE | chr12 | 25245351 | C>T | 14801 | 2 | 0.014 | NO |
| 05NE | chr12 | 25245348 | C>T | 14846 | 2 | 0.013 | NO |
| 74NE | chr12 | 25245347 | C>A | 22625 | 3 | 0.013 | NO |
| 72NE | chr12 | 25245347 | C>A | 7654 | 1 | 0.013 | NO |
| 74NE | chr12 | 25245349 | A>G | 22984 | 3 | 0.013 | NO |
| 72NE | chr12 | 25245349 | AC>A | 7707 | 1 | 0.013 | NO |
| 07NE | chr12 | 25245348 | C>T | 15464 | 2 | 0.013 | NO |
| 07NE | chr12 | 25245349 | A>G | 15490 | 2 | 0.013 | NO |
| 23NE | chr12 | 25245349 | A>C | 31127 | 4 | 0.013 | NO |
| 04NE | chr12 | 25245351 | C>A | 15693 | 2 | 0.013 | NO |
| 86NE | chr12 | 25245347 | C>T | 7876 | 1 | 0.013 | NO |
| 04NE | chr12 | 25245349 | AC>A | 15778 | 2 | 0.013 | NO |
| 60NE | chr12 | 25245349 | A>G | 16376 | 2 | 0.012 | NO |
| 36NE | chr12 | 25245347 | C>A | 24729 | 3 | 0.012 | NO |
| 36NE | chr12 | 25245350 | C>T | 24877 | 3 | 0.012 | NO |
| 02NE | chr12 | 25245349 | A>G | 16603 | 2 | 0.012 | NO |
| 54NE | chr12 | 25245350 | C>A | 33824 | 4 | 0.012 | NO |
| 40NE | chr12 | 25245349 | A>G | 16969 | 2 | 0.012 | NO |
| 03NE | chr12 | 25245349 | A>G | 17215 | 2 | 0.012 | NO |
| 14NE | chr12 | 25245351 | C>A | 25851 | 3 | 0.012 | NO |
| 14NE | chr12 | 25245349 | A>T | 25874 | 3 | 0.012 | NO |
| 14NE | chr12 | 25245348 | C>A | 25877 | 3 | 0.012 | NO |
| 47NE | chr12 | 25245348 | C>T | 17475 | 2 | 0.011 | NO |
| 47NE | chr12 | 25245351 | C>T | 17475 | 2 | 0.011 | NO |
| 53NE | chr12 | 25245349 | A>G | 26241 | 3 | 0.011 | NO |
| 19NE | chr12 | 25245350 | C>T | 35092 | 4 | 0.011 | NO |
| 98NE | chr12 | 25245350 | C>T | 44338 | 5 | 0.011 | NO |
| 85NE | chr12 | 25245347 | C>A | 8927 | 1 | 0.011 | NO |
| 85NE | chr12 | 25245348 | C>T | 8970 | 1 | 0.011 | NO |
| 85NE | chr12 | 25245349 | A>C | 8974 | 1 | 0.011 | NO |
| 17NE | chr12 | 25245351 | C>T | 53936 | 6 | 0.011 | NO |
| 17NE | chr12 | 25245350 | C>T | 54025 | 6 | 0.011 | NO |
| 79NE | chr12 | 25245347 | C>A | 9172 | 1 | 0.011 | NO |
| 73NE | chr12 | 25245348 | C>A | 9177 | 1 | 0.011 | NO |
| 79NE | chr12 | 25245351 | C>A | 9198 | 1 | 0.011 | NO |
| 09NE | chr12 | 25245347 | C>A | 18467 | 2 | 0.011 | NO |
| 79NE | chr12 | 25245349 | A>G | 9302 | 1 | 0.011 | NO |
| 09NE | chr12 | 25245349 | AC>A | 18642 | 2 | 0.011 | NO |
| 38NE | chr12 | 25245348 | CA>C | 9604 | 1 | 0.010 | NO |
| 42NE | chr12 | 25245348 | C>G | 9725 | 1 | 0.010 | NO |
| 42NE | chr12 | 25245350 | C>A | 9748 | 1 | 0.010 | NO |
| 42NE | chr12 | 25245349 | AC>A | 9766 | 1 | 0.010 | NO |
| 81NE | chr12 | 25245347 | C>T | 9999 | 1 | 0.010 | NO |
| 44NE | chr12 | 25245351 | C>T | 10102 | 1 | 0.010 | NO |
| 44NE | chr12 | 25245350 | C>T | 10134 | 1 | 0.010 | NO |
| 44NE | chr12 | 25245349 | A>C | 10176 | 1 | 0.010 | NO |
| 81NE | chr12 | 25245350 | C>G | 10179 | 1 | 0.010 | NO |
| 81NE | chr12 | 25245349 | A>C | 10196 | 1 | 0.010 | NO |
| 55NE | chr12 | 25245351 | C>A | 20722 | 2 | 0.010 | NO |
| 23NE | chr12 | 25245348 | C>T | 31097 | 3 | 0.010 | NO |
| 50NE | chr12 | 25245347 | C>A | 31145 | 3 | 0.010 | NO |
| 26NE | chr12 | 25245349 | AC>A | 21485 | 2 | 0.009 | NO |
| 61NE | chr12 | 25245349 | A>G | 10833 | 1 | 0.009 | NO |
| 98NE | chr12 | 25245347 | C>A | 44225 | 4 | 0.009 | NO |
| 98NE | chr12 | 25245348 | C>T | 44328 | 4 | 0.009 | NO |
| 06NE | chr12 | 25245351 | C>T | 11133 | 1 | 0.009 | NO |
| 54NE | chr12 | 25245351 | C>A | 33562 | 3 | 0.009 | NO |
| 64NE | chr12 | 25245348 | C>G | 34071 | 3 | 0.009 | NO |
| 56NE | chr12 | 25245348 | C>A | 11369 | 1 | 0.009 | NO |
| 56NE | chr12 | 25245349 | AC>A | 11403 | 1 | 0.009 | NO |
| 41NE | chr12 | 25245347 | C>T | 11490 | 1 | 0.009 | NO |
| 41NE | chr12 | 25245351 | C>A | 11496 | 1 | 0.009 | NO |
| 87NE | chr12 | 25245351 | C>T | 11505 | 1 | 0.009 | NO |
| 87NE | chr12 | 25245349 | A>G | 11585 | 1 | 0.009 | NO |
| 16NE | chr12 | 25245348 | C>T | 46367 | 4 | 0.009 | NO |
| 49NE | chr12 | 25245347 | C>A | 11789 | 1 | 0.008 | NO |
| 97NE | chr12 | 25245348 | C>T | 11818 | 1 | 0.008 | NO |
| 49NE | chr12 | 25245351 | C>A | 11828 | 1 | 0.008 | NO |
| 49NE | chr12 | 25245348 | C>T | 11840 | 1 | 0.008 | NO |
| 49NE | chr12 | 25245350 | C>T | 11844 | 1 | 0.008 | NO |
| 93NE | chr12 | 25245347 | C>A | 11896 | 1 | 0.008 | NO |
| 93NE | chr12 | 25245348 | C>T | 11931 | 1 | 0.008 | NO |
| 37NE | chr12 | 25245347 | C>A | 11971 | 1 | 0.008 | NO |
| 37NE | chr12 | 25245351 | C>T | 12012 | 1 | 0.008 | NO |
| 37NE | chr12 | 25245348 | C>T | 12034 | 1 | 0.008 | NO |
| 51NE | chr12 | 25245348 | C>T | 24531 | 2 | 0.008 | NO |
| 51NE | chr12 | 25245349 | A>G | 24659 | 2 | 0.008 | NO |
| 82NE | chr12 | 25245347 | C>A | 12874 | 1 | 0.008 | NO |
| 14NE | chr12 | 25245350 | C>A | 25892 | 2 | 0.008 | NO |
| 53NE | chr12 | 25245351 | C>T | 26102 | 2 | 0.008 | NO |
| 92NE | chr12 | 25245351 | C>T | 13156 | 1 | 0.008 | NO |
| 01NE | chr12 | 25245347 | C>A | 13174 | 1 | 0.008 | NO |
| 01NE | chr12 | 25245351 | C>A | 13192 | 1 | 0.008 | NO |
| 59NE | chr12 | 25245347 | C>A | 26501 | 2 | 0.008 | NO |
| 01NE | chr12 | 25245348 | C>T | 13281 | 1 | 0.008 | NO |
| 01NE | chr12 | 25245349 | AC>A | 13281 | 1 | 0.008 | NO |
| 43NE | chr12 | 25245350 | C>A | 13359 | 1 | 0.007 | NO |
| 43NE | chr12 | 25245349 | AC>A | 13364 | 1 | 0.007 | NO |
| 92NE | chr12 | 25245350 | C>A | 13401 | 1 | 0.007 | NO |
| 34NE | chr12 | 25245349 | A>C | 27263 | 2 | 0.007 | NO |
| 34NE | chr12 | 25245350 | C>T | 27282 | 2 | 0.007 | NO |
| 25NE | chr12 | 25245347 | C>T | 13668 | 1 | 0.007 | NO |
| 25NE | chr12 | 25245351 | C>A | 13668 | 1 | 0.007 | NO |
| 25NE | chr12 | 25245348 | CA>C | 13673 | 1 | 0.007 | NO |
| 57NE | chr12 | 25245348 | C>G | 27985 | 2 | 0.007 | NO |
| 77NE | chr12 | 25245348 | C>G | 14098 | 1 | 0.007 | NO |
| 57NE | chr12 | 25245350 | C>A | 28217 | 2 | 0.007 | NO |
| 57NE | chr12 | 25245349 | A>T | 28247 | 2 | 0.007 | NO |
| 24NE | chr12 | 25245347 | C>A | 14464 | 1 | 0.007 | NO |
| 24NE | chr12 | 25245348 | C>A | 14496 | 1 | 0.007 | NO |
| 58NE | chr12 | 25245347 | C>A | 29121 | 2 | 0.007 | NO |
| 58NE | chr12 | 25245351 | C>T | 29137 | 2 | 0.007 | NO |
| 48NE | chr12 | 25245347 | C>G | 14591 | 1 | 0.007 | NO |
| 48NE | chr12 | 25245351 | C>A | 14648 | 1 | 0.007 | NO |
| 48NE | chr12 | 25245350 | C>T | 14679 | 1 | 0.007 | NO |
| 05NE | chr12 | 25245347 | C>A | 14729 | 1 | 0.007 | NO |
| 98NE | chr12 | 25245351 | C>A | 44265 | 3 | 0.007 | NO |
| 05NE | chr12 | 25245351 | C>A | 14766 | 1 | 0.007 | NO |
| 80NE | chr12 | 25245349 | A>C | 14939 | 1 | 0.007 | NO |
| 80NE | chr12 | 25245350 | C>A | 14941 | 1 | 0.007 | NO |
| 08NE | chr12 | 25245347 | C>A | 15024 | 1 | 0.007 | NO |
| 90NE | chr12 | 25245348 | C>G | 15063 | 1 | 0.007 | NO |
| 08NE | chr12 | 25245349 | A>G | 15144 | 1 | 0.007 | NO |
| 07NE | chr12 | 25245350 | C>A | 15493 | 1 | 0.006 | NO |
| 88NE | chr12 | 25245347 | C>A | 31054 | 2 | 0.006 | NO |
| 88NE | chr12 | 25245348 | C>T | 31263 | 2 | 0.006 | NO |
| 50NE | chr12 | 25245351 | C>T | 31265 | 2 | 0.006 | NO |
| 04NE | chr12 | 25245347 | C>A | 15663 | 1 | 0.006 | NO |
| 50NE | chr12 | 25245348 | C>A | 31356 | 2 | 0.006 | NO |
| 04NE | chr12 | 25245348 | C>G | 15747 | 1 | 0.006 | NO |
| 60NE | chr12 | 25245351 | C>A | 16238 | 1 | 0.006 | NO |
| 02NE | chr12 | 25245351 | C>A | 16510 | 1 | 0.006 | NO |
| 45NE | chr12 | 25245347 | C>T | 16633 | 1 | 0.006 | NO |
| 54NE | chr12 | 25245347 | C>T | 33532 | 2 | 0.006 | NO |
| 65NE | chr12 | 25245348 | C>G | 16792 | 1 | 0.006 | NO |
| 65NE | chr12 | 25245349 | A>T | 16831 | 1 | 0.006 | NO |
| 54NE | chr12 | 25245348 | C>A | 33678 | 2 | 0.006 | NO |
| 40NE | chr12 | 25245348 | C>T | 16855 | 1 | 0.006 | NO |
| 62NE | chr12 | 25245347 | C>A | 16904 | 1 | 0.006 | NO |
| 54NE | chr12 | 25245349 | A>T | 33810 | 2 | 0.006 | NO |
| 40NE | chr12 | 25245350 | CCA>C | 16930 | 1 | 0.006 | NO |
| 64NE | chr12 | 25245349 | A>G | 34104 | 2 | 0.006 | NO |
| 62NE | chr12 | 25245350 | C>T | 17061 | 1 | 0.006 | NO |
| 89NE | chr12 | 25245351 | C>T | 17246 | 1 | 0.006 | NO |
| 89NE | chr12 | 25245347 | C>A | 17453 | 1 | 0.006 | NO |
| 19NE | chr12 | 25245347 | C>A | 34970 | 2 | 0.006 | NO |
| 89NE | chr12 | 25245348 | C>T | 17494 | 1 | 0.006 | NO |
| 17NE | chr12 | 25245347 | C>A | 53881 | 3 | 0.006 | NO |
| 94NE | chr12 | 25245347 | C>A | 18399 | 1 | 0.005 | NO |
| 94NE | chr12 | 25245348 | C>A | 18431 | 1 | 0.005 | NO |
| 09NE | chr12 | 25245348 | C>A | 18619 | 1 | 0.005 | NO |
| 91NE | chr12 | 25245351 | C>A | 19552 | 1 | 0.005 | NO |
| 91NE | chr12 | 25245348 | C>A | 19890 | 1 | 0.005 | NO |
| 55NE | chr12 | 25245347 | C>T | 20723 | 1 | 0.005 | NO |
| 55NE | chr12 | 25245350 | C>G | 21010 | 1 | 0.005 | NO |
| 55NE | chr12 | 25245349 | A>G | 21048 | 1 | 0.005 | NO |
| 10NE | chr12 | 25245347 | C>A | 22848 | 1 | 0.004 | NO |
| 10NE | chr12 | 25245348 | CA>C | 23035 | 1 | 0.004 | NO |
| 16NE | chr12 | 25245347 | C>A | 46268 | 2 | 0.004 | NO |
| 16NE | chr12 | 25245350 | C>T | 46375 | 2 | 0.004 | NO |
| 51NE | chr12 | 25245347 | C>A | 24369 | 1 | 0.004 | NO |
| 51NE | chr12 | 25245351 | C>A | 24443 | 1 | 0.004 | NO |
| 53NE | chr12 | 25245347 | C>A | 26038 | 1 | 0.004 | NO |
| 53NE | chr12 | 25245348 | C>T | 26146 | 1 | 0.004 | NO |
| 53NE | chr12 | 25245350 | C>A | 26273 | 1 | 0.004 | NO |
| 59NE | chr12 | 25245348 | C>T | 26645 | 1 | 0.004 | NO |
| 59NE | chr12 | 25245349 | AC>A | 26917 | 1 | 0.004 | NO |
| 34NE | chr12 | 25245347 | C>T | 27158 | 1 | 0.004 | NO |
| 57NE | chr12 | 25245347 | C>A | 27787 | 1 | 0.004 | NO |
| 57NE | chr12 | 25245351 | C>T | 27857 | 1 | 0.004 | NO |
| 58NE | chr12 | 25245350 | C>T | 29552 | 1 | 0.003 | NO |
| 23NE | chr12 | 25245350 | C>A | 31128 | 1 | 0.003 | NO |
| 50NE | chr12 | 25245349 | A>C | 31565 | 1 | 0.003 | NO |
| 64NE | chr12 | 25245347 | CCA>C | 33781 | 1 | 0.003 | NO |
| 02NE | chr12 | 25245347 | C>_ | 16476 | 0 | 0.000 | NO |
| 03NE | chr12 | 25245347 | C>_ | 17066 | 0 | 0.000 | NO |
| 03NE | chr12 | 25245348 | C>_ | 17205 | 0 | 0.000 | NO |
| 06NE | chr12 | 25245347 | C>_ | 11127 | 0 | 0.000 | NO |
| 07NE | chr12 | 25245347 | C>_ | 15380 | 0 | 0.000 | NO |
| 08NE | chr12 | 25245348 | C>_ | 15158 | 0 | 0.000 | NO |
| 11NE | chr12 | 25245347 | C>_ | 3828 | 0 | 0.000 | NO |
| 12NE | chr12 | 25245347 | C>_ | 6441 | 0 | 0.000 | NO |
| 12NE | chr12 | 25245348 | C>_ | 6476 | 0 | 0.000 | NO |
| 12NE | chr12 | 25245349 | A>_ | 6485 | 0 | 0.000 | NO |
| 12NE | chr12 | 25245351 | C>_ | 6454 | 0 | 0.000 | NO |
| 13NE | chr12 | 25245350 | C>_ | 3847 | 0 | 0.000 | NO |
| 15NE | chr12 | 25245350 | C>_ | 3484 | 0 | 0.000 | NO |
| 15NE | chr12 | 25245351 | C>_ | 3514 | 0 | 0.000 | NO |
| 18NE | chr12 | 25245350 | C>_ | 4107 | 0 | 0.000 | NO |
| 18NE | chr12 | 25245351 | C>_ | 4158 | 0 | 0.000 | NO |
| 20NE | chr12 | 25245350 | C>_ | 4234 | 0 | 0.000 | NO |
| 21NE | chr12 | 25245350 | C>_ | 3855 | 0 | 0.000 | NO |
| 26NE | chr12 | 25245347 | C>_ | 21411 | 0 | 0.000 | NO |
| 27NE | chr12 | 25245347 | C>_ | 13016 | 0 | 0.000 | NO |
| 28NE | chr12 | 25245347 | C>_ | 1118 | 0 | 0.000 | NO |
| 28NE | chr12 | 25245349 | A>_ | 1125 | 0 | 0.000 | NO |
| 28NE | chr12 | 25245351 | C>_ | 1118 | 0 | 0.000 | NO |
| 29NE | chr12 | 25245347 | C>_ | 2094 | 0 | 0.000 | NO |
| 29NE | chr12 | 25245348 | C>_ | 2103 | 0 | 0.000 | NO |
| 29NE | chr12 | 25245351 | C>_ | 2094 | 0 | 0.000 | NO |
| 30NE | chr12 | 25245347 | C>_ | 7352 | 0 | 0.000 | NO |
| 31NE | chr12 | 25245347 | C>_ | 6512 | 0 | 0.000 | NO |
| 31NE | chr12 | 25245348 | C>_ | 6532 | 0 | 0.000 | NO |
| 31NE | chr12 | 25245351 | C>_ | 6505 | 0 | 0.000 | NO |
| 32NE | chr12 | 25245347 | C>_ | 4293 | 0 | 0.000 | NO |
| 32NE | chr12 | 25245348 | C>_ | 4304 | 0 | 0.000 | NO |
| 32NE | chr12 | 25245349 | A>_ | 4304 | 0 | 0.000 | NO |
| 32NE | chr12 | 25245351 | C>_ | 4288 | 0 | 0.000 | NO |
| 33NE | chr12 | 25245348 | C>_ | 4381 | 0 | 0.000 | NO |
| 35NE | chr12 | 25245347 | C>_ | 11025 | 0 | 0.000 | NO |
| 35NE | chr12 | 25245348 | C>_ | 11061 | 0 | 0.000 | NO |
| 35NE | chr12 | 25245350 | C>_ | 11094 | 0 | 0.000 | NO |
| 35NE | chr12 | 25245351 | C>_ | 11066 | 0 | 0.000 | NO |
| 38NE | chr12 | 25245347 | C>_ | 9551 | 0 | 0.000 | NO |
| 41NE | chr12 | 25245349 | A>_ | 11529 | 0 | 0.000 | NO |
| 41NE | chr12 | 25245350 | C>_ | 11535 | 0 | 0.000 | NO |
| 42NE | chr12 | 25245347 | C>_ | 9664 | 0 | 0.000 | NO |
| 42NE | chr12 | 25245351 | C>_ | 9715 | 0 | 0.000 | NO |
| 43NE | chr12 | 25245351 | C>_ | 13332 | 0 | 0.000 | NO |
| 45NE | chr12 | 25245351 | C>_ | 16695 | 0 | 0.000 | NO |
| 46NE | chr12 | 25245347 | C>_ | 13801 | 0 | 0.000 | NO |
| 46NE | chr12 | 25245351 | C>_ | 13862 | 0 | 0.000 | NO |
| 47NE | chr12 | 25245347 | C>_ | 17411 | 0 | 0.000 | NO |
| 47NE | chr12 | 25245349 | A>_ | 17500 | 0 | 0.000 | NO |
| 51NE | chr12 | 25245350 | C>_ | 24679 | 0 | 0.000 | NO |
| 52NE | chr12 | 25245347 | C>_ | 1524 | 0 | 0.000 | NO |
| 52NE | chr12 | 25245350 | C>_ | 1542 | 0 | 0.000 | NO |
| 52NE | chr12 | 25245351 | C>_ | 1523 | 0 | 0.000 | NO |
| 60NE | chr12 | 25245347 | C>_ | 16232 | 0 | 0.000 | NO |
| 61NE | chr12 | 25245347 | C>_ | 10769 | 0 | 0.000 | NO |
| 61NE | chr12 | 25245348 | C>_ | 10797 | 0 | 0.000 | NO |
| 62NE | chr12 | 25245348 | C>_ | 17032 | 0 | 0.000 | NO |
| 63NE | chr12 | 25245348 | C>_ | 13560 | 0 | 0.000 | NO |
| 65NE | chr12 | 25245350 | C>_ | 16814 | 0 | 0.000 | NO |
| 56NE | chr12 | 25245347 | C>_ | 11358 | 0 | 0.000 | NO |
| 56NE | chr12 | 25245351 | C>_ | 11345 | 0 | 0.000 | NO |
| 66NE | chr12 | 25245347 | C>_ | 4322 | 0 | 0.000 | NO |
| 66NE | chr12 | 25245348 | C>_ | 4324 | 0 | 0.000 | NO |
| 67NE | chr12 | 25245347 | C>_ | 2968 | 0 | 0.000 | NO |
| 67NE | chr12 | 25245349 | A>_ | 2981 | 0 | 0.000 | NO |
| 68NE | chr12 | 25245347 | C>_ | 5499 | 0 | 0.000 | NO |
| 68NE | chr12 | 25245351 | C>_ | 5501 | 0 | 0.000 | NO |
| 69NE | chr12 | 25245348 | C>_ | 6289 | 0 | 0.000 | NO |
| 69NE | chr12 | 25245351 | C>_ | 6277 | 0 | 0.000 | NO |
| 70NE | chr12 | 25245347 | C>_ | 3187 | 0 | 0.000 | NO |
| 70NE | chr12 | 25245348 | C>_ | 3193 | 0 | 0.000 | NO |
| 71NE | chr12 | 25245347 | C>_ | 5312 | 0 | 0.000 | NO |
| 71NE | chr12 | 25245348 | C>_ | 5334 | 0 | 0.000 | NO |
| 71NE | chr12 | 25245351 | C>_ | 5316 | 0 | 0.000 | NO |
| 72NE | chr12 | 25245348 | C>_ | 7680 | 0 | 0.000 | NO |
| 72NE | chr12 | 25245350 | C>_ | 7716 | 0 | 0.000 | NO |
| 73NE | chr12 | 25245349 | A>_ | 9292 | 0 | 0.000 | NO |
| 74NE | chr12 | 25245348 | C>_ | 22711 | 0 | 0.000 | NO |
| 75NE | chr12 | 25245347 | C>_ | 9498 | 0 | 0.000 | NO |
| 75NE | chr12 | 25245348 | C>_ | 9547 | 0 | 0.000 | NO |
| 75NE | chr12 | 25245349 | A>_ | 9666 | 0 | 0.000 | NO |
| 76NE | chr12 | 25245349 | A>_ | 7115 | 0 | 0.000 | NO |
| 77NE | chr12 | 25245350 | C>_ | 14113 | 0 | 0.000 | NO |
| 77NE | chr12 | 25245351 | C>_ | 14084 | 0 | 0.000 | NO |
| 78NE | chr12 | 25245347 | C>_ | 3313 | 0 | 0.000 | NO |
| 78NE | chr12 | 25245348 | C>_ | 3322 | 0 | 0.000 | NO |
| 79NE | chr12 | 25245348 | C>_ | 9233 | 0 | 0.000 | NO |
| 80NE | chr12 | 25245348 | C>_ | 14812 | 0 | 0.000 | NO |
| 81NE | chr12 | 25245348 | C>_ | 10065 | 0 | 0.000 | NO |
| 82NE | chr12 | 25245348 | C>_ | 12932 | 0 | 0.000 | NO |
| 82NE | chr12 | 25245351 | C>_ | 12932 | 0 | 0.000 | NO |
| 83NE | chr12 | 25245347 | C>_ | 5196 | 0 | 0.000 | NO |
| 83NE | chr12 | 25245348 | C>_ | 5217 | 0 | 0.000 | NO |
| 84NE | chr12 | 25245347 | C>_ | 5108 | 0 | 0.000 | NO |
| 84NE | chr12 | 25245348 | C>_ | 5120 | 0 | 0.000 | NO |
| 86NE | chr12 | 25245349 | A>_ | 7931 | 0 | 0.000 | NO |
| 86NE | chr12 | 25245350 | C>_ | 7926 | 0 | 0.000 | NO |
| 87NE | chr12 | 25245347 | C>_ | 11494 | 0 | 0.000 | NO |
| 90NE | chr12 | 25245347 | C>_ | 15007 | 0 | 0.000 | NO |
| 90NE | chr12 | 25245351 | C>_ | 14788 | 0 | 0.000 | NO |
| 92NE | chr12 | 25245347 | C>_ | 13341 | 0 | 0.000 | NO |
| 93NE | chr12 | 25245350 | C>_ | 11946 | 0 | 0.000 | NO |
| 95NE | chr12 | 25245347 | C>_ | 6708 | 0 | 0.000 | NO |
| 96NE | chr12 | 25245347 | C>_ | 18418 | 0 | 0.000 | NO |
| ^1^Somatic mut, somatic mutation calls were described in Supplementary method. | | | | | | |  |

| **Table S5. Summary of targeted deep sequencing read information for *PIK3CA* mutation analyses.** | | | | | | | |
| --- | --- | --- | --- | --- | --- | --- | --- |
| ID | Chr | Position | Ref>Mut | Total reads | Mutation reads | VAF (%) | Somatic mut^1^ |
| 75NE | chr3 | 179234297 | A>G | 41838 | 864 | 2.065 | YES |
| 83NE | chr3 | 179234297 | A>G | 9657 | 174 | 1.802 | YES |
| 42NE | chr3 | 179234297 | A>G | 69177 | 709 | 1.025 | YES |
| 79NE | chr3 | 179234297 | A>G | 185773 | 975 | 0.525 | YES |
| 52NE | chr3 | 179234297 | A>G | 207392 | 945 | 0.456 | YES |
| 37NE | chr3 | 179234297 | A>G | 69475 | 274 | 0.394 | YES |
| 57NE | chr3 | 179234297 | A>T | 174226 | 676 | 0.388 | YES |
| 71NE | chr3 | 179234298 | T>A | 37818 | 144 | 0.381 | YES |
| 21NE | chr3 | 179234297 | A>G | 38135 | 144 | 0.378 | YES |
| 39NE | chr3 | 179234298 | T>G | 100447 | 372 | 0.370 | YES |
| 39NE | chr3 | 179234296 | C>T | 100797 | 354 | 0.351 | YES |
| 08NE | chr3 | 179234297 | A>G | 84133 | 281 | 0.334 | YES |
| 87NE | chr3 | 179234297 | A>G | 56446 | 173 | 0.306 | YES |
| 48NE | chr3 | 179234297 | A>G | 68296 | 202 | 0.296 | YES |
| 76NE | chr3 | 179234297 | A>G | 46031 | 120 | 0.261 | YES |
| 66NE | chr3 | 179234297 | A>T | 5612 | 12 | 0.214 | YES |
| 52NE | chr3 | 179234296 | C>G | 207247 | 393 | 0.190 | YES |
| 51NE | chr3 | 179234297 | A>T | 230416 | 412 | 0.179 | YES |
| 58NE | chr3 | 179234297 | A>G | 187331 | 332 | 0.177 | YES |
| 94NE | chr3 | 179234297 | A>G | 60711 | 107 | 0.176 | YES |
| 40NE | chr3 | 179234297 | A>G | 109141 | 185 | 0.170 | YES |
| 77NE | chr3 | 179234297 | A>G | 41331 | 54 | 0.131 | NO |
| 09NE | chr3 | 179234296 | C>T | 109279 | 125 | 0.114 | NO |
| 38NE | chr3 | 179234297 | A>G | 68411 | 75 | 0.110 | NO |
| 70NE | chr3 | 179234296 | C>T | 41640 | 45 | 0.108 | NO |
| 84NE | chr3 | 179234296 | C>T | 65048 | 68 | 0.105 | NO |
| 32NE | chr3 | 179234297 | A>G | 26898 | 28 | 0.104 | NO |
| 88NE | chr3 | 179234296 | C>T | 183657 | 185 | 0.101 | NO |
| 67NE | chr3 | 179234297 | A>C | 10029 | 9 | 0.090 | NO |
| 44NE | chr3 | 179234297 | A>G | 42707 | 36 | 0.084 | NO |
| 33NE | chr3 | 179234297 | A>C | 15661 | 13 | 0.083 | NO |
| 43NE | chr3 | 179234297 | A>G | 54814 | 41 | 0.075 | NO |
| 39NE | chr3 | 179234297 | A>G | 101293 | 74 | 0.073 | NO |
| 28NE | chr3 | 179234297 | A>C | 40164 | 29 | 0.072 | NO |
| 82NE | chr3 | 179234297 | A>G | 77177 | 49 | 0.063 | NO |
| 27NE | chr3 | 179234297 | A>C | 44953 | 27 | 0.060 | NO |
| 24NE | chr3 | 179234297 | A>C | 48294 | 29 | 0.060 | NO |
| 13NE | chr3 | 179234297 | A>G | 40383 | 22 | 0.054 | NO |
| 31NE | chr3 | 179234297 | A>C | 38985 | 21 | 0.054 | NO |
| 90NE | chr3 | 179234298 | T>G | 56846 | 30 | 0.053 | NO |
| 65NE | chr3 | 179234296 | C>A | 76182 | 40 | 0.053 | NO |
| 10NE | chr3 | 179234296 | C>A | 154327 | 80 | 0.052 | NO |
| 86NE | chr3 | 179234296 | C>A | 118766 | 60 | 0.051 | NO |
| 56NE | chr3 | 179234297 | A>C | 36441 | 18 | 0.049 | NO |
| 85NE | chr3 | 179234296 | C>A | 61601 | 30 | 0.049 | NO |
| 63NE | chr3 | 179234296 | C>A | 70211 | 34 | 0.048 | NO |
| 02NE | chr3 | 179234296 | C>A | 79982 | 38 | 0.048 | NO |
| 11NE | chr3 | 179234296 | C>A | 107642 | 51 | 0.047 | NO |
| 08NE | chr3 | 179234296 | C>A | 84575 | 40 | 0.047 | NO |
| 25NE | chr3 | 179234297 | A>C | 29670 | 14 | 0.047 | NO |
| 61NE | chr3 | 179234297 | A>G | 70488 | 33 | 0.047 | NO |
| 12NE | chr3 | 179234296 | C>A | 191823 | 87 | 0.045 | NO |
| 61NE | chr3 | 179234296 | C>A | 70750 | 32 | 0.045 | NO |
| 64NE | chr3 | 179234296 | C>A | 169094 | 74 | 0.044 | NO |
| 01NE | chr3 | 179234296 | C>A | 61197 | 26 | 0.042 | NO |
| 05NE | chr3 | 179234296 | C>A | 83433 | 35 | 0.042 | NO |
| 04NE | chr3 | 179234296 | C>A | 86427 | 36 | 0.042 | NO |
| 30NE | chr3 | 179234297 | A>C | 33734 | 14 | 0.042 | NO |
| 29NE | chr3 | 179234297 | A>C | 29539 | 12 | 0.041 | NO |
| 60NE | chr3 | 179234296 | C>A | 77162 | 31 | 0.040 | NO |
| 62NE | chr3 | 179234296 | C>A | 71236 | 27 | 0.038 | NO |
| 32NE | chr3 | 179234296 | C>A | 26964 | 10 | 0.037 | NO |
| 66NE | chr3 | 179234296 | C>A | 5640 | 2 | 0.035 | NO |
| 03NE | chr3 | 179234296 | C>A | 72428 | 25 | 0.035 | NO |
| 26NE | chr3 | 179234297 | A>C | 41940 | 14 | 0.033 | NO |
| 19NE | chr3 | 179234297 | A>C | 228122 | 75 | 0.033 | NO |
| 81NE | chr3 | 179234296 | C>T | 168866 | 55 | 0.033 | NO |
| 86NE | chr3 | 179234297 | A>G | 118306 | 38 | 0.032 | NO |
| 04NE | chr3 | 179234297 | A>G | 86058 | 27 | 0.031 | NO |
| 07NE | chr3 | 179234296 | C>A | 78414 | 24 | 0.031 | NO |
| 87NE | chr3 | 179234296 | C>A | 56689 | 17 | 0.030 | NO |
| 60NE | chr3 | 179234297 | A>G | 76922 | 23 | 0.030 | NO |
| 67NE | chr3 | 179234298 | T>C | 10038 | 3 | 0.030 | NO |
| 01NE | chr3 | 179234297 | A>G | 60982 | 18 | 0.030 | NO |
| 10NE | chr3 | 179234297 | A>G | 153763 | 45 | 0.029 | NO |
| 11NE | chr3 | 179234297 | A>G | 107160 | 31 | 0.029 | NO |
| 09NE | chr3 | 179234297 | A>G | 108809 | 31 | 0.028 | NO |
| 06NE | chr3 | 179234296 | C>A | 79728 | 22 | 0.028 | NO |
| 63NE | chr3 | 179234297 | A>G | 69899 | 19 | 0.027 | NO |
| 07NE | chr3 | 179234297 | A>G | 78040 | 21 | 0.027 | NO |
| 65NE | chr3 | 179234297 | A>G | 75979 | 20 | 0.026 | NO |
| 85NE | chr3 | 179234297 | A>G | 61322 | 16 | 0.026 | NO |
| 17NE | chr3 | 179234296 | C>T | 143469 | 37 | 0.026 | NO |
| 12NE | chr3 | 179234297 | A>G | 191119 | 48 | 0.025 | NO |
| 02NE | chr3 | 179234297 | A>G | 79678 | 20 | 0.025 | NO |
| 19NE | chr3 | 179234298 | T>C | 227377 | 57 | 0.025 | NO |
| 92NE | chr3 | 179234296 | C>A | 40010 | 10 | 0.025 | NO |
| 03NE | chr3 | 179234297 | A>G | 72066 | 18 | 0.025 | NO |
| 78NE | chr3 | 179234297 | A>G | 57326 | 14 | 0.024 | NO |
| 70NE | chr3 | 179234298 | T>C | 41524 | 10 | 0.024 | NO |
| 26NE | chr3 | 179234296 | C>A | 42058 | 10 | 0.024 | NO |
| 64NE | chr3 | 179234297 | A>G | 168370 | 40 | 0.024 | NO |
| 29NE | chr3 | 179234296 | C>A | 29634 | 7 | 0.024 | NO |
| 96NE | chr3 | 179234296 | C>T | 56002 | 13 | 0.023 | NO |
| 50NE | chr3 | 179234296 | C>A | 274874 | 63 | 0.023 | NO |
| 24NE | chr3 | 179234298 | T>C | 48374 | 11 | 0.023 | NO |
| 06NE | chr3 | 179234297 | A>G | 79399 | 18 | 0.023 | NO |
| 18NE | chr3 | 179234297 | A>G | 35756 | 8 | 0.022 | NO |
| 91NE | chr3 | 179234298 | T>G | 76822 | 17 | 0.022 | NO |
| 74NE | chr3 | 179234297 | A>G | 38819 | 8 | 0.021 | NO |
| 20NE | chr3 | 179234297 | A>G | 39216 | 8 | 0.020 | NO |
| 62NE | chr3 | 179234297 | A>G | 70989 | 14 | 0.020 | NO |
| 93NE | chr3 | 179234298 | T>G | 46531 | 9 | 0.019 | NO |
| 88NE | chr3 | 179234297 | A>G | 182931 | 35 | 0.019 | NO |
| 48NE | chr3 | 179234298 | T>C | 68041 | 13 | 0.019 | NO |
| 14NE | chr3 | 179234298 | T>C | 217678 | 41 | 0.019 | NO |
| 34NE | chr3 | 179234297 | A>G | 98131 | 18 | 0.018 | NO |
| 14NE | chr3 | 179234297 | A>C | 218026 | 39 | 0.018 | NO |
| 16NE | chr3 | 179234297 | A>G | 259527 | 46 | 0.018 | NO |
| 97NE | chr3 | 179234297 | A>T | 67793 | 12 | 0.018 | NO |
| 98NE | chr3 | 179234298 | T>C | 195867 | 34 | 0.017 | NO |
| 77NE | chr3 | 179234296 | C>T | 41285 | 7 | 0.017 | NO |
| 23NE | chr3 | 179234298 | T>A | 252883 | 42 | 0.017 | NO |
| 16NE | chr3 | 179234298 | T>C | 259133 | 43 | 0.017 | NO |
| 89NE | chr3 | 179234298 | T>C | 66843 | 11 | 0.016 | NO |
| 22NE | chr3 | 179234297 | A>C | 30692 | 5 | 0.016 | NO |
| 15NE | chr3 | 179234296 | C>T | 32251 | 5 | 0.016 | NO |
| 20NE | chr3 | 179234298 | T>C | 38840 | 6 | 0.015 | NO |
| 17NE | chr3 | 179234297 | A>G | 142785 | 22 | 0.015 | NO |
| 35NE | chr3 | 179234296 | C>T | 46676 | 7 | 0.015 | NO |
| 92NE | chr3 | 179234298 | T>A | 40192 | 6 | 0.015 | NO |
| 97NE | chr3 | 179234296 | C>T | 67052 | 10 | 0.015 | NO |
| 92NE | chr3 | 179234297 | A>G | 40357 | 6 | 0.015 | NO |
| 97NE | chr3 | 179234298 | T>C | 67540 | 10 | 0.015 | NO |
| 98NE | chr3 | 179234296 | C>T | 196890 | 29 | 0.015 | NO |
| 13NE | chr3 | 179234296 | C>A | 40958 | 6 | 0.015 | NO |
| 98NE | chr3 | 179234297 | A>G | 196097 | 28 | 0.014 | NO |
| 95NE | chr3 | 179234298 | T>A | 63182 | 9 | 0.014 | NO |
| 96NE | chr3 | 179234298 | T>G | 56405 | 8 | 0.014 | NO |
| 96NE | chr3 | 179234297 | A>G | 56565 | 8 | 0.014 | NO |
| 17NE | chr3 | 179234298 | T>C | 142692 | 20 | 0.014 | NO |
| 40NE | chr3 | 179234298 | T>A | 108058 | 15 | 0.014 | NO |
| 56NE | chr3 | 179234296 | C>T | 36547 | 5 | 0.014 | NO |
| 25NE | chr3 | 179234296 | C>A | 29736 | 4 | 0.013 | NO |
| 27NE | chr3 | 179234296 | C>A | 45111 | 6 | 0.013 | NO |
| 73NE | chr3 | 179234296 | C>T | 37705 | 5 | 0.013 | NO |
| 94NE | chr3 | 179234298 | T>G | 60445 | 8 | 0.013 | NO |
| 22NE | chr3 | 179234298 | T>A | 30239 | 4 | 0.013 | NO |
| 76NE | chr3 | 179234296 | C>T | 45958 | 6 | 0.013 | NO |
| 23NE | chr3 | 179234297 | A>C | 253089 | 33 | 0.013 | NO |
| 51NE | chr3 | 179234296 | C>T | 230196 | 30 | 0.013 | NO |
| 22NE | chr3 | 179234296 | C>T | 31120 | 4 | 0.013 | NO |
| 47NE | chr3 | 179234296 | C>T | 93393 | 12 | 0.013 | NO |
| 93NE | chr3 | 179234297 | A>T | 46697 | 6 | 0.013 | NO |
| 31NE | chr3 | 179234296 | C>A | 39089 | 5 | 0.013 | NO |
| 33NE | chr3 | 179234296 | C>A | 15715 | 2 | 0.013 | NO |
| 41NE | chr3 | 179234298 | T>C | 95187 | 12 | 0.013 | NO |
| 23NE | chr3 | 179234296 | C>T | 254087 | 32 | 0.013 | NO |
| 45NE | chr3 | 179234296 | C>T | 95357 | 12 | 0.013 | NO |
| 15NE | chr3 | 179234297 | A>G | 31794 | 4 | 0.013 | NO |
| 13NE | chr3 | 179234298 | T>C | 39807 | 5 | 0.013 | NO |
| 28NE | chr3 | 179234296 | C>A | 40345 | 5 | 0.012 | NO |
| 90NE | chr3 | 179234296 | C>A | 56516 | 7 | 0.012 | NO |
| 84NE | chr3 | 179234298 | T>C | 65409 | 8 | 0.012 | NO |
| 68NE | chr3 | 179234298 | T>C | 131307 | 16 | 0.012 | NO |
| 05NE | chr3 | 179234297 | A>G | 83069 | 10 | 0.012 | NO |
| 89NE | chr3 | 179234296 | C>T | 66458 | 8 | 0.012 | NO |
| 75NE | chr3 | 179234296 | C>A | 41660 | 5 | 0.012 | NO |
| 05NE | chr3 | 179234298 | T>C | 83417 | 10 | 0.012 | NO |
| 70NE | chr3 | 179234297 | A>G | 41718 | 5 | 0.012 | NO |
| 30NE | chr3 | 179234298 | T>A | 33755 | 4 | 0.012 | NO |
| 30NE | chr3 | 179234296 | C>A | 33846 | 4 | 0.012 | NO |
| 44NE | chr3 | 179234298 | T>A | 42383 | 5 | 0.012 | NO |
| 42NE | chr3 | 179234298 | T>C | 68629 | 8 | 0.012 | NO |
| 69NE | chr3 | 179234296 | C>T | 43512 | 5 | 0.011 | NO |
| 85NE | chr3 | 179234298 | T>C | 61538 | 7 | 0.011 | NO |
| 61NE | chr3 | 179234298 | T>C | 70699 | 8 | 0.011 | NO |
| 34NE | chr3 | 179234296 | C>T | 98024 | 11 | 0.011 | NO |
| 46NE | chr3 | 179234298 | T>C | 71365 | 8 | 0.011 | NO |
| 56NE | chr3 | 179234298 | T>C | 36456 | 4 | 0.011 | NO |
| 14NE | chr3 | 179234296 | C>T | 218866 | 24 | 0.011 | NO |
| 47NE | chr3 | 179234298 | T>C | 93093 | 10 | 0.011 | NO |
| 08NE | chr3 | 179234298 | T>A | 84537 | 9 | 0.011 | NO |
| 68NE | chr3 | 179234296 | C>T | 131568 | 14 | 0.011 | NO |
| 36NE | chr3 | 179234298 | T>C | 94120 | 10 | 0.011 | NO |
| 50NE | chr3 | 179234297 | A>G | 275061 | 29 | 0.011 | NO |
| 90NE | chr3 | 179234297 | A>G | 57094 | 6 | 0.011 | NO |
| 78NE | chr3 | 179234296 | C>A | 57303 | 6 | 0.010 | NO |
| 74NE | chr3 | 179234298 | T>C | 38448 | 4 | 0.010 | NO |
| 80NE | chr3 | 179234296 | C>T | 183595 | 19 | 0.010 | NO |
| 83NE | chr3 | 179234296 | C>A | 9677 | 1 | 0.010 | NO |
| 24NE | chr3 | 179234296 | C>A | 48461 | 5 | 0.010 | NO |
| 34NE | chr3 | 179234298 | T>A | 97745 | 10 | 0.010 | NO |
| 94NE | chr3 | 179234296 | C>T | 60120 | 6 | 0.010 | NO |
| 16NE | chr3 | 179234296 | C>T | 260549 | 26 | 0.010 | NO |
| 67NE | chr3 | 179234296 | C>A | 10061 | 1 | 0.010 | NO |
| 10NE | chr3 | 179234298 | T>A | 154142 | 15 | 0.010 | NO |
| 79NE | chr3 | 179234298 | T>C | 185365 | 18 | 0.010 | NO |
| 77NE | chr3 | 179234298 | T>A | 41244 | 4 | 0.010 | NO |
| 15NE | chr3 | 179234298 | T>C | 31466 | 3 | 0.010 | NO |
| 44NE | chr3 | 179234296 | C>T | 42539 | 4 | 0.009 | NO |
| 11NE | chr3 | 179234298 | T>C | 107483 | 10 | 0.009 | NO |
| 80NE | chr3 | 179234297 | A>G | 183807 | 17 | 0.009 | NO |
| 59NE | chr3 | 179234297 | A>G | 173369 | 16 | 0.009 | NO |
| 69NE | chr3 | 179234298 | T>A | 43390 | 4 | 0.009 | NO |
| 72NE | chr3 | 179234298 | T>C | 43456 | 4 | 0.009 | NO |
| 57NE | chr3 | 179234296 | C>T | 173920 | 16 | 0.009 | NO |
| 69NE | chr3 | 179234297 | A>T | 43559 | 4 | 0.009 | NO |
| 43NE | chr3 | 179234296 | C>A | 54569 | 5 | 0.009 | NO |
| 49NE | chr3 | 179234297 | A>G | 87847 | 8 | 0.009 | NO |
| 07NE | chr3 | 179234298 | T>C | 78353 | 7 | 0.009 | NO |
| 55NE | chr3 | 179234296 | C>T | 157592 | 14 | 0.009 | NO |
| 55NE | chr3 | 179234297 | A>G | 157824 | 14 | 0.009 | NO |
| 54NE | chr3 | 179234298 | T>C | 192044 | 17 | 0.009 | NO |
| 38NE | chr3 | 179234298 | T>C | 68114 | 6 | 0.009 | NO |
| 19NE | chr3 | 179234296 | C>A | 228896 | 20 | 0.009 | NO |
| 35NE | chr3 | 179234297 | A>G | 46702 | 4 | 0.009 | NO |
| 58NE | chr3 | 179234298 | T>C | 186944 | 16 | 0.009 | NO |
| 18NE | chr3 | 179234298 | T>C | 35312 | 3 | 0.008 | NO |
| 46NE | chr3 | 179234296 | C>T | 71639 | 6 | 0.008 | NO |
| 03NE | chr3 | 179234298 | T>A | 72380 | 6 | 0.008 | NO |
| 18NE | chr3 | 179234296 | C>T | 36242 | 3 | 0.008 | NO |
| 59NE | chr3 | 179234298 | T>C | 172875 | 14 | 0.008 | NO |
| 59NE | chr3 | 179234296 | C>A | 173095 | 14 | 0.008 | NO |
| 53NE | chr3 | 179234298 | T>C | 226437 | 18 | 0.008 | NO |
| 51NE | chr3 | 179234298 | T>C | 230098 | 18 | 0.008 | NO |
| 60NE | chr3 | 179234298 | T>C | 77162 | 6 | 0.008 | NO |
| 21NE | chr3 | 179234296 | C>T | 38678 | 3 | 0.008 | NO |
| 31NE | chr3 | 179234298 | T>A | 38992 | 3 | 0.008 | NO |
| 55NE | chr3 | 179234298 | T>C | 157361 | 12 | 0.008 | NO |
| 84NE | chr3 | 179234297 | A>C | 65641 | 5 | 0.008 | NO |
| 20NE | chr3 | 179234296 | C>A | 39821 | 3 | 0.008 | NO |
| 06NE | chr3 | 179234298 | T>C | 79653 | 6 | 0.008 | NO |
| 53NE | chr3 | 179234296 | C>T | 226634 | 17 | 0.008 | NO |
| 09NE | chr3 | 179234298 | T>C | 109080 | 8 | 0.007 | NO |
| 41NE | chr3 | 179234297 | A>G | 95837 | 7 | 0.007 | NO |
| 26NE | chr3 | 179234298 | T>A | 41982 | 3 | 0.007 | NO |
| 57NE | chr3 | 179234298 | T>C | 173684 | 12 | 0.007 | NO |
| 29NE | chr3 | 179234298 | T>A | 29520 | 2 | 0.007 | NO |
| 52NE | chr3 | 179234298 | T>A | 207041 | 14 | 0.007 | NO |
| 86NE | chr3 | 179234298 | T>A | 118643 | 8 | 0.007 | NO |
| 25NE | chr3 | 179234298 | T>C | 29719 | 2 | 0.007 | NO |
| 65NE | chr3 | 179234298 | T>C | 76095 | 5 | 0.007 | NO |
| 91NE | chr3 | 179234296 | C>T | 76389 | 5 | 0.007 | NO |
| 01NE | chr3 | 179234298 | T>C | 61164 | 4 | 0.007 | NO |
| 64NE | chr3 | 179234298 | T>C | 168984 | 11 | 0.007 | NO |
| 82NE | chr3 | 179234298 | T>C | 76823 | 5 | 0.007 | NO |
| 58NE | chr3 | 179234296 | C>A | 187092 | 12 | 0.006 | NO |
| 36NE | chr3 | 179234297 | A>G | 94454 | 6 | 0.006 | NO |
| 81NE | chr3 | 179234298 | T>A | 168690 | 10 | 0.006 | NO |
| 04NE | chr3 | 179234298 | T>A | 86418 | 5 | 0.006 | NO |
| 37NE | chr3 | 179234298 | T>A | 69202 | 4 | 0.006 | NO |
| 37NE | chr3 | 179234296 | C>A | 69405 | 4 | 0.006 | NO |
| 53NE | chr3 | 179234297 | A>G | 226764 | 13 | 0.006 | NO |
| 49NE | chr3 | 179234296 | C>T | 87802 | 5 | 0.006 | NO |
| 46NE | chr3 | 179234297 | A>G | 71908 | 4 | 0.006 | NO |
| 40NE | chr3 | 179234296 | C>A | 108414 | 6 | 0.006 | NO |
| 80NE | chr3 | 179234298 | T>C | 183349 | 10 | 0.005 | NO |
| 88NE | chr3 | 179234298 | T>A | 183526 | 10 | 0.005 | NO |
| 21NE | chr3 | 179234298 | T>A | 37543 | 2 | 0.005 | NO |
| 73NE | chr3 | 179234298 | T>A | 37586 | 2 | 0.005 | NO |
| 36NE | chr3 | 179234296 | C>T | 94362 | 5 | 0.005 | NO |
| 73NE | chr3 | 179234297 | A>T | 37897 | 2 | 0.005 | NO |
| 45NE | chr3 | 179234298 | T>C | 94844 | 5 | 0.005 | NO |
| 71NE | chr3 | 179234296 | C>A | 37973 | 2 | 0.005 | NO |
| 71NE | chr3 | 179234297 | A>G | 38054 | 2 | 0.005 | NO |
| 12NE | chr3 | 179234298 | T>C | 191702 | 10 | 0.005 | NO |
| 82NE | chr3 | 179234296 | C>T | 77070 | 4 | 0.005 | NO |
| 74NE | chr3 | 179234296 | C>A | 38602 | 2 | 0.005 | NO |
| 91NE | chr3 | 179234297 | A>G | 77208 | 4 | 0.005 | NO |
| 02NE | chr3 | 179234298 | T>A | 79958 | 4 | 0.005 | NO |
| 28NE | chr3 | 179234298 | T>C | 40276 | 2 | 0.005 | NO |
| 75NE | chr3 | 179234298 | T>A | 41441 | 2 | 0.005 | NO |
| 95NE | chr3 | 179234296 | C>G | 62777 | 3 | 0.005 | NO |
| 95NE | chr3 | 179234297 | A>G | 63398 | 3 | 0.005 | NO |
| 72NE | chr3 | 179234296 | C>A | 43590 | 2 | 0.005 | NO |
| 72NE | chr3 | 179234297 | A>G | 43640 | 2 | 0.005 | NO |
| 49NE | chr3 | 179234298 | T>C | 87552 | 4 | 0.005 | NO |
| 68NE | chr3 | 179234297 | A>G | 131708 | 6 | 0.005 | NO |
| 89NE | chr3 | 179234297 | A>G | 67093 | 3 | 0.004 | NO |
| 38NE | chr3 | 179234296 | C>T | 68318 | 3 | 0.004 | NO |
| 42NE | chr3 | 179234296 | C>G | 68924 | 3 | 0.004 | NO |
| 93NE | chr3 | 179234296 | C>T | 46271 | 2 | 0.004 | NO |
| 79NE | chr3 | 179234296 | C>T | 185556 | 8 | 0.004 | NO |
| 41NE | chr3 | 179234296 | C>T | 95506 | 4 | 0.004 | NO |
| 45NE | chr3 | 179234297 | A>G | 95794 | 4 | 0.004 | NO |
| 54NE | chr3 | 179234297 | A>G | 192265 | 8 | 0.004 | NO |
| 43NE | chr3 | 179234298 | T>C | 54335 | 2 | 0.004 | NO |
| 81NE | chr3 | 179234297 | A>T | 169103 | 6 | 0.004 | NO |
| 87NE | chr3 | 179234298 | T>G | 56656 | 2 | 0.004 | NO |
| 78NE | chr3 | 179234298 | T>C | 57138 | 2 | 0.004 | NO |
| 54NE | chr3 | 179234296 | C>T | 192116 | 6 | 0.003 | NO |
| 48NE | chr3 | 179234296 | C>T | 68226 | 2 | 0.003 | NO |
| 50NE | chr3 | 179234298 | T>C | 274575 | 8 | 0.003 | NO |
| 62NE | chr3 | 179234298 | T>A | 71188 | 2 | 0.003 | NO |
| 27NE | chr3 | 179234298 | T>G | 45010 | 1 | 0.002 | NO |
| 35NE | chr3 | 179234298 | T>A | 46562 | 1 | 0.002 | NO |
| 47NE | chr3 | 179234297 | A>G | 93482 | 2 | 0.002 | NO |
| 63NE | chr3 | 179234298 | T>C | 70193 | 1 | 0.001 | NO |
| 32NE | chr3 | 179234298 | T>_ | 26902 | 0 | 0.000 | NO |
| 33NE | chr3 | 179234298 | T>_ | 15677 | 0 | 0.000 | NO |
| 66NE | chr3 | 179234298 | T>_ | 5620 | 0 | 0.000 | NO |
| 76NE | chr3 | 179234298 | T>_ | 45902 | 0 | 0.000 | NO |
| 83NE | chr3 | 179234298 | T>_ | 9657 | 0 | 0.000 | NO |
| ^1^Somatic mutation, somatic mutation calls were described in Supplementary method. | | | | | | | |

| **Table S6. Summary of targeted deep sequencing read information for *PPP2R1A* mutation analyses.** | | | | | | | |
| --- | --- | --- | --- | --- | --- | --- | --- |
| ID | Chr | Position | Ref>Mut | Total reads | Mutation reads | VAF (%) | Somatic mut^1^ |
| 58NE | chr19 | 52212718 | C>G | 53220 | 1214 | 2.281 | YES |
| 51NE | chr19 | 52212729 | C>T | 41482 | 480 | 1.157 | YES |
| 50NE | chr19 | 52212718 | C>A | 54421 | 593 | 1.090 | YES |
| 72NE | chr19 | 52212729 | C>T | 49830 | 490 | 0.983 | YES |
| 72NE | chr19 | 52212726 | C>T | 49758 | 477 | 0.959 | YES |
| 82NE | chr19 | 52212726 | C>T | 6815 | 55 | 0.807 | YES |
| 98NE | chr19 | 52212730 | G>A | 2431 | 19 | 0.782 | YES |
| 51NE | chr19 | 52212726 | C>T | 41736 | 321 | 0.769 | YES |
| 42NE | chr19 | 52212729 | C>T | 7146 | 52 | 0.728 | YES |
| 83NE | chr19 | 52212718 | C>G | 9587 | 63 | 0.657 | YES |
| 45NE | chr19 | 52212727 | G>A | 13627 | 64 | 0.470 | YES |
| 40NE | chr19 | 52212718 | C>G | 13146 | 49 | 0.373 | YES |
| 44NE | chr19 | 52212730 | G>A | 8544 | 20 | 0.234 | YES |
| 47NE | chr19 | 52212729 | C>T | 15493 | 34 | 0.219 | YES |
| 43NE | chr19 | 52212730 | G>A | 10829 | 22 | 0.203 | YES |
| 90NE | chr19 | 52212729 | C>T | 19603 | 34 | 0.173 | YES |
| 10NE | chr19 | 52212730 | G>A | 5041 | 8 | 0.159 | YES |
| 84NE | chr19 | 52212729 | C>T | 19866 | 27 | 0.136 | NO |
| 83NE | chr19 | 52212729 | C>T | 9541 | 12 | 0.126 | NO |
| 09NE | chr19 | 52212729 | C>T | 5656 | 7 | 0.124 | NO |
| 64NE | chr19 | 52212729 | C>T | 14194 | 16 | 0.113 | NO |
| 76NE | chr19 | 52212726 | C>T | 11026 | 12 | 0.109 | NO |
| 07NE | chr19 | 52212726 | C>T | 4262 | 4 | 0.094 | NO |
| 87NE | chr19 | 52212730 | G>A | 5549 | 5 | 0.090 | NO |
| 05NE | chr19 | 52212726 | C>T | 3467 | 3 | 0.087 | NO |
| 22NE | chr19 | 52212727 | G>A | 4845 | 4 | 0.083 | NO |
| 24NE | chr19 | 52212726 | C>T | 30323 | 25 | 0.082 | NO |
| 91NE | chr19 | 52212726 | C>T | 9714 | 8 | 0.082 | NO |
| 14NE | chr19 | 52212729 | C>T | 41286 | 32 | 0.078 | NO |
| 11NE | chr19 | 52212726 | C>T | 5380 | 4 | 0.074 | NO |
| 02NE | chr19 | 52212729 | C>T | 4056 | 3 | 0.074 | NO |
| 58NE | chr19 | 52212729 | C>T | 51641 | 38 | 0.074 | NO |
| 25NE | chr19 | 52212726 | C>T | 21954 | 16 | 0.073 | NO |
| 66NE | chr19 | 52212727 | G>A | 5497 | 4 | 0.073 | NO |
| 20NE | chr19 | 52212726 | C>T | 5506 | 4 | 0.073 | NO |
| 66NE | chr19 | 52212726 | C>T | 5508 | 4 | 0.073 | NO |
| 86NE | chr19 | 52212730 | G>A | 2869 | 2 | 0.070 | NO |
| 86NE | chr19 | 52212729 | C>T | 2898 | 2 | 0.069 | NO |
| 23NE | chr19 | 52212730 | G>A | 64484 | 44 | 0.068 | NO |
| 69NE | chr19 | 52212730 | G>A | 23078 | 15 | 0.065 | NO |
| 12NE | chr19 | 52212730 | G>T | 3148 | 2 | 0.064 | NO |
| 39NE | chr19 | 52212729 | C>T | 7882 | 5 | 0.063 | NO |
| 22NE | chr19 | 52212729 | C>T | 4852 | 3 | 0.062 | NO |
| 32NE | chr19 | 52212726 | C>T | 9804 | 6 | 0.061 | NO |
| 30NE | chr19 | 52212727 | G>A | 19883 | 12 | 0.060 | NO |
| 93NE | chr19 | 52212727 | G>A | 1681 | 1 | 0.059 | NO |
| 18NE | chr19 | 52212726 | C>T | 6728 | 4 | 0.059 | NO |
| 93NE | chr19 | 52212729 | CG>C | 1687 | 1 | 0.059 | NO |
| 21NE | chr19 | 52212729 | C>T | 6766 | 4 | 0.059 | NO |
| 04NE | chr19 | 52212730 | G>A | 3412 | 2 | 0.059 | NO |
| 65NE | chr19 | 52212729 | C>T | 3414 | 2 | 0.059 | NO |
| 77NE | chr19 | 52212727 | G>A | 17439 | 10 | 0.057 | NO |
| 01NE | chr19 | 52212729 | C>T | 3528 | 2 | 0.057 | NO |
| 64NE | chr19 | 52212726 | C>T | 14214 | 8 | 0.056 | NO |
| 67NE | chr19 | 52212726 | C>T | 10663 | 6 | 0.056 | NO |
| 24NE | chr19 | 52212727 | G>A | 30342 | 17 | 0.056 | NO |
| 11NE | chr19 | 52212729 | C>T | 5356 | 3 | 0.056 | NO |
| 04NE | chr19 | 52212727 | G>A | 3577 | 2 | 0.056 | NO |
| 60NE | chr19 | 52212727 | G>A | 18037 | 10 | 0.055 | NO |
| 83NE | chr19 | 52212730 | G>A | 9439 | 5 | 0.053 | NO |
| 68NE | chr19 | 52212729 | C>T | 59102 | 31 | 0.052 | NO |
| 89NE | chr19 | 52212726 | C>T | 9555 | 5 | 0.052 | NO |
| 32NE | chr19 | 52212730 | G>A | 9748 | 5 | 0.051 | NO |
| 23NE | chr19 | 52212727 | G>A | 64562 | 33 | 0.051 | NO |
| 74NE | chr19 | 52212729 | C>T | 48921 | 25 | 0.051 | NO |
| 39NE | chr19 | 52212726 | C>T | 7858 | 4 | 0.051 | NO |
| 26NE | chr19 | 52212730 | G>A | 24005 | 12 | 0.050 | NO |
| 29NE | chr19 | 52212718 | C>A | 16158 | 8 | 0.050 | NO |
| 02NE | chr19 | 52212730 | G>A | 4085 | 2 | 0.049 | NO |
| 03NE | chr19 | 52212726 | C>T | 4143 | 2 | 0.048 | NO |
| 03NE | chr19 | 52212727 | G>A | 4178 | 2 | 0.048 | NO |
| 71NE | chr19 | 52212726 | C>T | 84270 | 40 | 0.047 | NO |
| 07NE | chr19 | 52212727 | G>A | 4249 | 2 | 0.047 | NO |
| 67NE | chr19 | 52212729 | C>T | 10673 | 5 | 0.047 | NO |
| 07NE | chr19 | 52212730 | G>A | 4295 | 2 | 0.047 | NO |
| 13NE | chr19 | 52212726 | C>T | 6505 | 3 | 0.046 | NO |
| 24NE | chr19 | 52212729 | C>T | 30409 | 14 | 0.046 | NO |
| 13NE | chr19 | 52212730 | G>A | 6539 | 3 | 0.046 | NO |
| 43NE | chr19 | 52212729 | C>T | 10908 | 5 | 0.046 | NO |
| 77NE | chr19 | 52212718 | C>A | 17483 | 8 | 0.046 | NO |
| 76NE | chr19 | 52212727 | G>T | 11033 | 5 | 0.045 | NO |
| 27NE | chr19 | 52212718 | C>A | 31414 | 14 | 0.045 | NO |
| 68NE | chr19 | 52212730 | G>A | 58459 | 26 | 0.044 | NO |
| 82NE | chr19 | 52212730 | G>A | 6817 | 3 | 0.044 | NO |
| 82NE | chr19 | 52212727 | G>A | 6837 | 3 | 0.044 | NO |
| 75NE | chr19 | 52212729 | C>T | 6936 | 3 | 0.043 | NO |
| 16NE | chr19 | 52212727 | G>A | 53771 | 23 | 0.043 | NO |
| 19NE | chr19 | 52212730 | G>A | 63320 | 27 | 0.043 | NO |
| 42NE | chr19 | 52212730 | G>A | 7085 | 3 | 0.042 | NO |
| 83NE | chr19 | 52212727 | G>T | 9530 | 4 | 0.042 | NO |
| 58NE | chr19 | 52212730 | G>A | 50763 | 21 | 0.041 | NO |
| 98NE | chr19 | 52212727 | G>A | 2433 | 1 | 0.041 | NO |
| 16NE | chr19 | 52212730 | G>A | 53696 | 22 | 0.041 | NO |
| 22NE | chr19 | 52212730 | G>A | 4897 | 2 | 0.041 | NO |
| 32NE | chr19 | 52212727 | G>T | 9814 | 4 | 0.041 | NO |
| 73NE | chr19 | 52212726 | C>T | 66809 | 27 | 0.040 | NO |
| 10NE | chr19 | 52212727 | G>A | 4971 | 2 | 0.040 | NO |
| 57NE | chr19 | 52212729 | C>T | 50878 | 20 | 0.039 | NO |
| 23NE | chr19 | 52212726 | C>T | 64408 | 25 | 0.039 | NO |
| 47NE | chr19 | 52212726 | C>T | 15490 | 6 | 0.039 | NO |
| 60NE | chr19 | 52212726 | C>T | 18117 | 7 | 0.039 | NO |
| 58NE | chr19 | 52212727 | G>A | 51814 | 20 | 0.039 | NO |
| 71NE | chr19 | 52212730 | G>A | 83786 | 32 | 0.038 | NO |
| 19NE | chr19 | 52212727 | G>A | 63529 | 24 | 0.038 | NO |
| 29NE | chr19 | 52212727 | G>T | 16058 | 6 | 0.037 | NO |
| 26NE | chr19 | 52212718 | C>A | 24225 | 9 | 0.037 | NO |
| 31NE | chr19 | 52212718 | C>A | 16367 | 6 | 0.037 | NO |
| 11NE | chr19 | 52212718 | C>A | 5460 | 2 | 0.037 | NO |
| 20NE | chr19 | 52212727 | G>A | 5493 | 2 | 0.036 | NO |
| 25NE | chr19 | 52212729 | C>T | 21981 | 8 | 0.036 | NO |
| 20NE | chr19 | 52212729 | C>T | 5498 | 2 | 0.036 | NO |
| 09NE | chr19 | 52212726 | C>T | 5665 | 2 | 0.035 | NO |
| 49NE | chr19 | 52212729 | C>T | 11337 | 4 | 0.035 | NO |
| 30NE | chr19 | 52212729 | C>T | 19904 | 7 | 0.035 | NO |
| 09NE | chr19 | 52212730 | G>A | 5699 | 2 | 0.035 | NO |
| 15NE | chr19 | 52212727 | G>A | 5747 | 2 | 0.035 | NO |
| 15NE | chr19 | 52212729 | C>T | 5768 | 2 | 0.035 | NO |
| 36NE | chr19 | 52212726 | C>T | 46360 | 16 | 0.035 | NO |
| 86NE | chr19 | 52212727 | G>T | 2948 | 1 | 0.034 | NO |
| 86NE | chr19 | 52212717 | C>T | 2984 | 1 | 0.034 | NO |
| 26NE | chr19 | 52212726 | C>T | 24100 | 8 | 0.033 | NO |
| 26NE | chr19 | 52212727 | G>A | 24116 | 8 | 0.033 | NO |
| 24NE | chr19 | 52212730 | G>A | 30154 | 10 | 0.033 | NO |
| 06NE | chr19 | 52212730 | G>A | 6066 | 2 | 0.033 | NO |
| 24NE | chr19 | 52212718 | C>A | 30438 | 10 | 0.033 | NO |
| 58NE | chr19 | 52212726 | C>T | 51950 | 17 | 0.033 | NO |
| 23NE | chr19 | 52212729 | C>T | 64452 | 21 | 0.033 | NO |
| 06NE | chr19 | 52212718 | C>A | 6140 | 2 | 0.033 | NO |
| 56NE | chr19 | 52212729 | C>T | 21811 | 7 | 0.032 | NO |
| 56NE | chr19 | 52212718 | C>A | 21847 | 7 | 0.032 | NO |
| 27NE | chr19 | 52212726 | C>T | 31295 | 10 | 0.032 | NO |
| 25NE | chr19 | 52212727 | G>T | 21935 | 7 | 0.032 | NO |
| 78NE | chr19 | 52212729 | C>T | 3140 | 1 | 0.032 | NO |
| 78NE | chr19 | 52212727 | G>T | 3147 | 1 | 0.032 | NO |
| 81NE | chr19 | 52212726 | C>T | 47215 | 15 | 0.032 | NO |
| 19NE | chr19 | 52212726 | C>T | 63239 | 20 | 0.032 | NO |
| 12NE | chr19 | 52212726 | C>T | 3162 | 1 | 0.032 | NO |
| 52NE | chr19 | 52212727 | G>A | 22154 | 7 | 0.032 | NO |
| 83NE | chr19 | 52212726 | C>T | 9523 | 3 | 0.032 | NO |
| 29NE | chr19 | 52212730 | G>T | 15883 | 5 | 0.031 | NO |
| 40NE | chr19 | 52212730 | G>A | 12753 | 4 | 0.031 | NO |
| 40NE | chr19 | 52212726 | C>T | 12892 | 4 | 0.031 | NO |
| 40NE | chr19 | 52212727 | G>A | 12915 | 4 | 0.031 | NO |
| 91NE | chr19 | 52212729 | C>T | 9714 | 3 | 0.031 | NO |
| 13NE | chr19 | 52212729 | C>T | 6482 | 2 | 0.031 | NO |
| 74NE | chr19 | 52212726 | C>T | 48818 | 15 | 0.031 | NO |
| 38NE | chr19 | 52212730 | G>T | 9792 | 3 | 0.031 | NO |
| 31NE | chr19 | 52212727 | G>T | 16330 | 5 | 0.031 | NO |
| 38NE | chr19 | 52212729 | C>T | 9807 | 3 | 0.031 | NO |
| 90NE | chr19 | 52212726 | C>T | 19633 | 6 | 0.031 | NO |
| 46NE | chr19 | 52212730 | G>A | 13093 | 4 | 0.031 | NO |
| 32NE | chr19 | 52212729 | C>T | 9821 | 3 | 0.031 | NO |
| 17NE | chr19 | 52212729 | C>T | 3284 | 1 | 0.030 | NO |
| 17NE | chr19 | 52212726 | C>A | 3285 | 1 | 0.030 | NO |
| 17NE | chr19 | 52212727 | G>A | 3293 | 1 | 0.030 | NO |
| 38NE | chr19 | 52212718 | C>G | 9884 | 3 | 0.030 | NO |
| 17NE | chr19 | 52212718 | C>A | 3301 | 1 | 0.030 | NO |
| 17NE | chr19 | 52212717 | C>A | 3304 | 1 | 0.030 | NO |
| 18NE | chr19 | 52212727 | G>A | 6681 | 2 | 0.030 | NO |
| 21NE | chr19 | 52212727 | G>A | 6747 | 2 | 0.030 | NO |
| 33NE | chr19 | 52212726 | C>T | 13593 | 4 | 0.029 | NO |
| 33NE | chr19 | 52212727 | G>T | 13595 | 4 | 0.029 | NO |
| 33NE | chr19 | 52212729 | C>T | 13628 | 4 | 0.029 | NO |
| 33NE | chr19 | 52212718 | C>A | 13632 | 4 | 0.029 | NO |
| 59NE | chr19 | 52212729 | C>T | 51174 | 15 | 0.029 | NO |
| 21NE | chr19 | 52212730 | G>A | 6841 | 2 | 0.029 | NO |
| 65NE | chr19 | 52212718 | C>T | 3461 | 1 | 0.029 | NO |
| 75NE | chr19 | 52212727 | G>T | 6929 | 2 | 0.029 | NO |
| 04NE | chr19 | 52212729 | C>T | 3466 | 1 | 0.029 | NO |
| 05NE | chr19 | 52212727 | G>A | 3467 | 1 | 0.029 | NO |
| 68NE | chr19 | 52212727 | G>A | 58995 | 17 | 0.029 | NO |
| 75NE | chr19 | 52212718 | C>A | 6949 | 2 | 0.029 | NO |
| 77NE | chr19 | 52212729 | C>T | 17434 | 5 | 0.029 | NO |
| 67NE | chr19 | 52212730 | G>T | 10559 | 3 | 0.028 | NO |
| 67NE | chr19 | 52212727 | G>A | 10660 | 3 | 0.028 | NO |
| 01NE | chr19 | 52212730 | G>A | 3555 | 1 | 0.028 | NO |
| 72NE | chr19 | 52212718 | C>A | 49779 | 14 | 0.028 | NO |
| 50NE | chr19 | 52212726 | C>T | 53437 | 15 | 0.028 | NO |
| 67NE | chr19 | 52212718 | C>A | 10718 | 3 | 0.028 | NO |
| 42NE | chr19 | 52212727 | G>A | 7161 | 2 | 0.028 | NO |
| 60NE | chr19 | 52212730 | G>A | 17979 | 5 | 0.028 | NO |
| 28NE | chr19 | 52212718 | C>A | 25400 | 7 | 0.028 | NO |
| 01NE | chr19 | 52212718 | C>A | 3640 | 1 | 0.027 | NO |
| 76NE | chr19 | 52212718 | C>A | 11056 | 3 | 0.027 | NO |
| 04NE | chr19 | 52212717 | C>A | 3719 | 1 | 0.027 | NO |
| 73NE | chr19 | 52212718 | C>A | 66983 | 18 | 0.027 | NO |
| 81NE | chr19 | 52212718 | C>A | 48397 | 13 | 0.027 | NO |
| 19NE | chr19 | 52212729 | C>T | 63394 | 17 | 0.027 | NO |
| 49NE | chr19 | 52212727 | G>A | 11332 | 3 | 0.026 | NO |
| 16NE | chr19 | 52212726 | C>T | 53621 | 14 | 0.026 | NO |
| 69NE | chr19 | 52212726 | C>T | 23224 | 6 | 0.026 | NO |
| 70NE | chr19 | 52212726 | C>T | 58114 | 15 | 0.026 | NO |
| 69NE | chr19 | 52212729 | C>T | 23273 | 6 | 0.026 | NO |
| 80NE | chr19 | 52212718 | C>A | 46808 | 12 | 0.026 | NO |
| 39NE | chr19 | 52212730 | G>A | 7856 | 2 | 0.025 | NO |
| 30NE | chr19 | 52212718 | C>A | 19955 | 5 | 0.025 | NO |
| 26NE | chr19 | 52212729 | C>T | 24177 | 6 | 0.025 | NO |
| 62NE | chr19 | 52212727 | G>A | 4037 | 1 | 0.025 | NO |
| 62NE | chr19 | 52212726 | C>T | 4048 | 1 | 0.025 | NO |
| 62NE | chr19 | 52212729 | C>T | 4056 | 1 | 0.025 | NO |
| 31NE | chr19 | 52212726 | C>T | 16296 | 4 | 0.025 | NO |
| 62NE | chr19 | 52212717 | C>A | 4074 | 1 | 0.025 | NO |
| 03NE | chr19 | 52212729 | C>T | 4082 | 1 | 0.024 | NO |
| 02NE | chr19 | 52212726 | C>G | 4104 | 1 | 0.024 | NO |
| 14NE | chr19 | 52212727 | G>A | 41356 | 10 | 0.024 | NO |
| 80NE | chr19 | 52212727 | G>A | 45561 | 11 | 0.024 | NO |
| 73NE | chr19 | 52212729 | C>T | 66954 | 16 | 0.024 | NO |
| 71NE | chr19 | 52212727 | G>T | 84276 | 20 | 0.024 | NO |
| 28NE | chr19 | 52212727 | G>T | 25287 | 6 | 0.024 | NO |
| 28NE | chr19 | 52212729 | C>T | 25306 | 6 | 0.024 | NO |
| 71NE | chr19 | 52212718 | C>A | 84386 | 20 | 0.024 | NO |
| 53NE | chr19 | 52212727 | G>T | 17253 | 4 | 0.023 | NO |
| 03NE | chr19 | 52212717 | C>A | 4318 | 1 | 0.023 | NO |
| 53NE | chr19 | 52212729 | C>T | 17272 | 4 | 0.023 | NO |
| 07NE | chr19 | 52212718 | C>T | 4337 | 1 | 0.023 | NO |
| 07NE | chr19 | 52212717 | C>T | 4341 | 1 | 0.023 | NO |
| 03NE | chr19 | 52212718 | C>A | 4344 | 1 | 0.023 | NO |
| 56NE | chr19 | 52212726 | C>T | 21761 | 5 | 0.023 | NO |
| 46NE | chr19 | 52212726 | C>T | 13224 | 3 | 0.023 | NO |
| 52NE | chr19 | 52212729 | C>T | 22127 | 5 | 0.023 | NO |
| 27NE | chr19 | 52212730 | G>T | 31121 | 7 | 0.022 | NO |
| 34NE | chr19 | 52212729 | C>T | 35568 | 8 | 0.022 | NO |
| 80NE | chr19 | 52212730 | G>A | 44536 | 10 | 0.022 | NO |
| 27NE | chr19 | 52212729 | C>T | 31376 | 7 | 0.022 | NO |
| 45NE | chr19 | 52212726 | C>T | 13577 | 3 | 0.022 | NO |
| 68NE | chr19 | 52212726 | C>T | 59012 | 13 | 0.022 | NO |
| 35NE | chr19 | 52212729 | C>T | 18158 | 4 | 0.022 | NO |
| 35NE | chr19 | 52212726 | C>T | 18160 | 4 | 0.022 | NO |
| 14NE | chr19 | 52212726 | C>A | 41219 | 9 | 0.022 | NO |
| 36NE | chr19 | 52212729 | C>T | 46355 | 10 | 0.022 | NO |
| 36NE | chr19 | 52212727 | G>A | 46470 | 10 | 0.022 | NO |
| 69NE | chr19 | 52212727 | G>T | 23252 | 5 | 0.022 | NO |
| 69NE | chr19 | 52212718 | C>A | 23277 | 5 | 0.021 | NO |
| 71NE | chr19 | 52212729 | C>T | 84429 | 18 | 0.021 | NO |
| 89NE | chr19 | 52212730 | G>T | 9465 | 2 | 0.021 | NO |
| 55NE | chr19 | 52212729 | C>T | 47369 | 10 | 0.021 | NO |
| 89NE | chr19 | 52212727 | G>A | 9491 | 2 | 0.021 | NO |
| 89NE | chr19 | 52212717 | C>T | 9617 | 2 | 0.021 | NO |
| 74NE | chr19 | 52212727 | G>T | 48840 | 10 | 0.020 | NO |
| 22NE | chr19 | 52212726 | C>T | 4887 | 1 | 0.020 | NO |
| 38NE | chr19 | 52212726 | C>T | 9780 | 2 | 0.020 | NO |
| 74NE | chr19 | 52212718 | C>A | 48955 | 10 | 0.020 | NO |
| 38NE | chr19 | 52212727 | G>A | 9804 | 2 | 0.020 | NO |
| 32NE | chr19 | 52212718 | C>A | 9844 | 2 | 0.020 | NO |
| 68NE | chr19 | 52212718 | C>A | 59110 | 12 | 0.020 | NO |
| 30NE | chr19 | 52212730 | G>T | 19758 | 4 | 0.020 | NO |
| 30NE | chr19 | 52212726 | C>T | 19857 | 4 | 0.020 | NO |
| 28NE | chr19 | 52212730 | G>A | 24985 | 5 | 0.020 | NO |
| 79NE | chr19 | 52212726 | C>T | 61466 | 12 | 0.020 | NO |
| 47NE | chr19 | 52212730 | G>A | 15467 | 3 | 0.019 | NO |
| 59NE | chr19 | 52212718 | C>G | 52776 | 10 | 0.019 | NO |
| 11NE | chr19 | 52212727 | G>A | 5336 | 1 | 0.019 | NO |
| 29NE | chr19 | 52212729 | C>T | 16061 | 3 | 0.019 | NO |
| 11NE | chr19 | 52212730 | G>A | 5384 | 1 | 0.019 | NO |
| 55NE | chr19 | 52212717 | C>A | 48721 | 9 | 0.018 | NO |
| 56NE | chr19 | 52212727 | G>T | 21778 | 4 | 0.018 | NO |
| 31NE | chr19 | 52212729 | C>T | 16336 | 3 | 0.018 | NO |
| 25NE | chr19 | 52212730 | G>T | 21786 | 4 | 0.018 | NO |
| 43NE | chr19 | 52212726 | C>T | 10932 | 2 | 0.018 | NO |
| 76NE | chr19 | 52212730 | G>T | 10970 | 2 | 0.018 | NO |
| 87NE | chr19 | 52212727 | G>A | 5495 | 1 | 0.018 | NO |
| 25NE | chr19 | 52212718 | C>A | 21994 | 4 | 0.018 | NO |
| 66NE | chr19 | 52212718 | C>A | 5527 | 1 | 0.018 | NO |
| 20NE | chr19 | 52212730 | G>A | 5537 | 1 | 0.018 | NO |
| 87NE | chr19 | 52212726 | C>T | 5537 | 1 | 0.018 | NO |
| 43NE | chr19 | 52212718 | C>A | 11120 | 2 | 0.018 | NO |
| 20NE | chr19 | 52212718 | C>A | 5586 | 1 | 0.018 | NO |
| 87NE | chr19 | 52212718 | C>A | 5617 | 1 | 0.018 | NO |
| 49NE | chr19 | 52212730 | G>A | 11335 | 2 | 0.018 | NO |
| 49NE | chr19 | 52212718 | C>A | 11356 | 2 | 0.018 | NO |
| 08NE | chr19 | 52212727 | G>A | 5680 | 1 | 0.018 | NO |
| 59NE | chr19 | 52212726 | C>T | 51385 | 9 | 0.018 | NO |
| 08NE | chr19 | 52212729 | CG>C | 5728 | 1 | 0.017 | NO |
| 53NE | chr19 | 52212730 | G>A | 17218 | 3 | 0.017 | NO |
| 15NE | chr19 | 52212726 | C>A | 5746 | 1 | 0.017 | NO |
| 77NE | chr19 | 52212730 | G>A | 17311 | 3 | 0.017 | NO |
| 15NE | chr19 | 52212730 | G>A | 5802 | 1 | 0.017 | NO |
| 88NE | chr19 | 52212727 | G>T | 5810 | 1 | 0.017 | NO |
| 08NE | chr19 | 52212717 | C>A | 5821 | 1 | 0.017 | NO |
| 70NE | chr19 | 52212729 | C>T | 58230 | 10 | 0.017 | NO |
| 88NE | chr19 | 52212729 | CG>C | 5847 | 1 | 0.017 | NO |
| 15NE | chr19 | 52212717 | C>A | 5854 | 1 | 0.017 | NO |
| 88NE | chr19 | 52212726 | C>T | 5874 | 1 | 0.017 | NO |
| 37NE | chr19 | 52212729 | C>T | 11775 | 2 | 0.017 | NO |
| 14NE | chr19 | 52212730 | G>A | 41291 | 7 | 0.017 | NO |
| 34NE | chr19 | 52212726 | C>T | 35562 | 6 | 0.017 | NO |
| 61NE | chr19 | 52212727 | G>A | 11873 | 2 | 0.017 | NO |
| 37NE | chr19 | 52212717 | C>A | 11884 | 2 | 0.017 | NO |
| 37NE | chr19 | 52212718 | C>A | 11886 | 2 | 0.017 | NO |
| 61NE | chr19 | 52212729 | C>T | 11889 | 2 | 0.017 | NO |
| 61NE | chr19 | 52212726 | C>T | 11890 | 2 | 0.017 | NO |
| 60NE | chr19 | 52212729 | CG>C | 18084 | 3 | 0.017 | NO |
| 35NE | chr19 | 52212727 | G>A | 18190 | 3 | 0.016 | NO |
| 73NE | chr19 | 52212727 | G>T | 66810 | 11 | 0.016 | NO |
| 79NE | chr19 | 52212729 | C>T | 61063 | 10 | 0.016 | NO |
| 72NE | chr19 | 52212730 | G>T | 49479 | 8 | 0.016 | NO |
| 72NE | chr19 | 52212727 | G>A | 49731 | 8 | 0.016 | NO |
| 28NE | chr19 | 52212726 | C>T | 25261 | 4 | 0.016 | NO |
| 57NE | chr19 | 52212727 | G>A | 51050 | 8 | 0.016 | NO |
| 80NE | chr19 | 52212729 | C>T | 45363 | 7 | 0.015 | NO |
| 90NE | chr19 | 52212730 | G>T | 19472 | 3 | 0.015 | NO |
| 90NE | chr19 | 52212727 | G>A | 19535 | 3 | 0.015 | NO |
| 36NE | chr19 | 52212730 | G>A | 46323 | 7 | 0.015 | NO |
| 73NE | chr19 | 52212730 | G>A | 66225 | 10 | 0.015 | NO |
| 50NE | chr19 | 52212729 | C>T | 53127 | 8 | 0.015 | NO |
| 13NE | chr19 | 52212717 | C>T | 6654 | 1 | 0.015 | NO |
| 18NE | chr19 | 52212729 | C>T | 6720 | 1 | 0.015 | NO |
| 45NE | chr19 | 52212730 | G>T | 13487 | 2 | 0.015 | NO |
| 18NE | chr19 | 52212730 | G>A | 6752 | 1 | 0.015 | NO |
| 33NE | chr19 | 52212730 | G>A | 13518 | 2 | 0.015 | NO |
| 21NE | chr19 | 52212726 | C>A | 6798 | 1 | 0.015 | NO |
| 45NE | chr19 | 52212729 | C>T | 13604 | 2 | 0.015 | NO |
| 82NE | chr19 | 52212729 | CG>C | 6829 | 1 | 0.015 | NO |
| 18NE | chr19 | 52212717 | C>A | 6843 | 1 | 0.015 | NO |
| 82NE | chr19 | 52212718 | C>T | 6878 | 1 | 0.015 | NO |
| 75NE | chr19 | 52212726 | C>T | 6923 | 1 | 0.014 | NO |
| 63NE | chr19 | 52212730 | G>A | 13897 | 2 | 0.014 | NO |
| 63NE | chr19 | 52212726 | C>T | 13972 | 2 | 0.014 | NO |
| 63NE | chr19 | 52212717 | C>A | 14070 | 2 | 0.014 | NO |
| 63NE | chr19 | 52212718 | C>A | 14072 | 2 | 0.014 | NO |
| 64NE | chr19 | 52212730 | G>A | 14151 | 2 | 0.014 | NO |
| 64NE | chr19 | 52212727 | G>A | 14154 | 2 | 0.014 | NO |
| 34NE | chr19 | 52212730 | G>A | 35534 | 5 | 0.014 | NO |
| 42NE | chr19 | 52212726 | C>A | 7138 | 1 | 0.014 | NO |
| 70NE | chr19 | 52212730 | G>A | 57794 | 8 | 0.014 | NO |
| 42NE | chr19 | 52212717 | C>A | 7274 | 1 | 0.014 | NO |
| 42NE | chr19 | 52212718 | C>A | 7276 | 1 | 0.014 | NO |
| 41NE | chr19 | 52212726 | CG>C | 7297 | 1 | 0.014 | NO |
| 41NE | chr19 | 52212727 | GGC>G | 7298 | 1 | 0.014 | NO |
| 41NE | chr19 | 52212729 | CG>C | 7308 | 1 | 0.014 | NO |
| 41NE | chr19 | 52212717 | C>A | 7320 | 1 | 0.014 | NO |
| 52NE | chr19 | 52212730 | G>A | 22081 | 3 | 0.014 | NO |
| 50NE | chr19 | 52212730 | G>A | 52584 | 7 | 0.013 | NO |
| 16NE | chr19 | 52212729 | C>T | 53676 | 7 | 0.013 | NO |
| 16NE | chr19 | 52212718 | C>A | 53917 | 7 | 0.013 | NO |
| 47NE | chr19 | 52212727 | G>A | 15503 | 2 | 0.013 | NO |
| 27NE | chr19 | 52212727 | G>T | 31286 | 4 | 0.013 | NO |
| 39NE | chr19 | 52212718 | C>A | 7888 | 1 | 0.013 | NO |
| 39NE | chr19 | 52212717 | C>T | 7895 | 1 | 0.013 | NO |
| 54NE | chr19 | 52212730 | G>A | 15979 | 2 | 0.013 | NO |
| 54NE | chr19 | 52212729 | C>A | 16028 | 2 | 0.012 | NO |
| 29NE | chr19 | 52212726 | C>T | 16039 | 2 | 0.012 | NO |
| 54NE | chr19 | 52212726 | C>T | 16072 | 2 | 0.012 | NO |
| 74NE | chr19 | 52212730 | G>T | 48393 | 6 | 0.012 | NO |
| 31NE | chr19 | 52212730 | G>T | 16208 | 2 | 0.012 | NO |
| 29NE | chr19 | 52212717 | C>T | 16240 | 2 | 0.012 | NO |
| 51NE | chr19 | 52212730 | G>T | 41043 | 5 | 0.012 | NO |
| 31NE | chr19 | 52212717 | C>T | 16425 | 2 | 0.012 | NO |
| 70NE | chr19 | 52212727 | G>A | 58141 | 7 | 0.012 | NO |
| 57NE | chr19 | 52212726 | C>T | 51184 | 6 | 0.012 | NO |
| 44NE | chr19 | 52212729 | C>T | 8635 | 1 | 0.012 | NO |
| 53NE | chr19 | 52212726 | C>A | 17298 | 2 | 0.012 | NO |
| 48NE | chr19 | 52212726 | C>T | 8696 | 1 | 0.011 | NO |
| 48NE | chr19 | 52212729 | C>T | 8696 | 1 | 0.011 | NO |
| 77NE | chr19 | 52212726 | C>T | 17401 | 2 | 0.011 | NO |
| 48NE | chr19 | 52212718 | C>A | 8764 | 1 | 0.011 | NO |
| 44NE | chr19 | 52212717 | C>A | 8767 | 1 | 0.011 | NO |
| 77NE | chr19 | 52212717 | C>T | 17555 | 2 | 0.011 | NO |
| 50NE | chr19 | 52212727 | G>T | 53196 | 6 | 0.011 | NO |
| 60NE | chr19 | 52212718 | C>A | 18248 | 2 | 0.011 | NO |
| 55NE | chr19 | 52212726 | C>T | 47594 | 5 | 0.011 | NO |
| 89NE | chr19 | 52212729 | C>G | 9540 | 1 | 0.010 | NO |
| 83NE | chr19 | 52212717 | C>A | 9628 | 1 | 0.010 | NO |
| 91NE | chr19 | 52212730 | G>A | 9648 | 1 | 0.010 | NO |
| 91NE | chr19 | 52212727 | G>A | 9692 | 1 | 0.010 | NO |
| 70NE | chr19 | 52212718 | C>A | 58225 | 6 | 0.010 | NO |
| 91NE | chr19 | 52212718 | C>A | 9794 | 1 | 0.010 | NO |
| 91NE | chr19 | 52212717 | C>T | 9799 | 1 | 0.010 | NO |
| 90NE | chr19 | 52212718 | C>T | 19776 | 2 | 0.010 | NO |
| 84NE | chr19 | 52212727 | G>A | 19783 | 2 | 0.010 | NO |
| 32NE | chr19 | 52212717 | C>T | 9892 | 1 | 0.010 | NO |
| 84NE | chr19 | 52212726 | C>T | 19875 | 2 | 0.010 | NO |
| 57NE | chr19 | 52212730 | G>A | 49954 | 5 | 0.010 | NO |
| 79NE | chr19 | 52212727 | G>T | 61190 | 6 | 0.010 | NO |
| 14NE | chr19 | 52212718 | C>A | 41470 | 4 | 0.010 | NO |
| 79NE | chr19 | 52212717 | C>A | 62575 | 6 | 0.010 | NO |
| 51NE | chr19 | 52212718 | C>A | 42577 | 4 | 0.009 | NO |
| 23NE | chr19 | 52212717 | C>A | 64681 | 6 | 0.009 | NO |
| 56NE | chr19 | 52212730 | G>A | 21644 | 2 | 0.009 | NO |
| 50NE | chr19 | 52212717 | C>A | 54304 | 5 | 0.009 | NO |
| 76NE | chr19 | 52212717 | C>A | 11111 | 1 | 0.009 | NO |
| 52NE | chr19 | 52212717 | C>G | 22290 | 2 | 0.009 | NO |
| 49NE | chr19 | 52212726 | C>T | 11315 | 1 | 0.009 | NO |
| 80NE | chr19 | 52212726 | C>T | 45592 | 4 | 0.009 | NO |
| 80NE | chr19 | 52212717 | C>A | 46713 | 4 | 0.009 | NO |
| 69NE | chr19 | 52212717 | C>T | 23372 | 2 | 0.009 | NO |
| 81NE | chr19 | 52212729 | C>T | 46971 | 4 | 0.009 | NO |
| 37NE | chr19 | 52212730 | G>A | 11756 | 1 | 0.009 | NO |
| 81NE | chr19 | 52212727 | G>T | 47115 | 4 | 0.008 | NO |
| 37NE | chr19 | 52212726 | C>T | 11787 | 1 | 0.008 | NO |
| 37NE | chr19 | 52212727 | G>A | 11812 | 1 | 0.008 | NO |
| 61NE | chr19 | 52212730 | G>A | 11844 | 1 | 0.008 | NO |
| 34NE | chr19 | 52212727 | G>A | 35676 | 3 | 0.008 | NO |
| 61NE | chr19 | 52212717 | C>A | 11956 | 1 | 0.008 | NO |
| 34NE | chr19 | 52212718 | C>A | 35881 | 3 | 0.008 | NO |
| 61NE | chr19 | 52212718 | C>A | 11965 | 1 | 0.008 | NO |
| 79NE | chr19 | 52212730 | G>A | 60255 | 5 | 0.008 | NO |
| 26NE | chr19 | 52212717 | C>T | 24290 | 2 | 0.008 | NO |
| 72NE | chr19 | 52212717 | C>A | 50018 | 4 | 0.008 | NO |
| 19NE | chr19 | 52212718 | C>T | 63920 | 5 | 0.008 | NO |
| 59NE | chr19 | 52212727 | G>A | 51291 | 4 | 0.008 | NO |
| 40NE | chr19 | 52212729 | CG>C | 12884 | 1 | 0.008 | NO |
| 57NE | chr19 | 52212717 | C>A | 52387 | 4 | 0.008 | NO |
| 57NE | chr19 | 52212718 | C>T | 52454 | 4 | 0.008 | NO |
| 46NE | chr19 | 52212729 | C>T | 13189 | 1 | 0.008 | NO |
| 46NE | chr19 | 52212727 | G>A | 13248 | 1 | 0.008 | NO |
| 46NE | chr19 | 52212717 | C>A | 13448 | 1 | 0.007 | NO |
| 46NE | chr19 | 52212718 | C>A | 13452 | 1 | 0.007 | NO |
| 16NE | chr19 | 52212717 | C>A | 53908 | 4 | 0.007 | NO |
| 14NE | chr19 | 52212717 | C>A | 41441 | 3 | 0.007 | NO |
| 45NE | chr19 | 52212717 | C>G | 13841 | 1 | 0.007 | NO |
| 45NE | chr19 | 52212718 | C>A | 13852 | 1 | 0.007 | NO |
| 51NE | chr19 | 52212727 | G>A | 41617 | 3 | 0.007 | NO |
| 63NE | chr19 | 52212727 | G>A | 13914 | 1 | 0.007 | NO |
| 63NE | chr19 | 52212729 | CG>C | 13951 | 1 | 0.007 | NO |
| 71NE | chr19 | 52212717 | C>T | 84710 | 6 | 0.007 | NO |
| 51NE | chr19 | 52212717 | C>A | 42509 | 3 | 0.007 | NO |
| 64NE | chr19 | 52212717 | C>T | 14283 | 1 | 0.007 | NO |
| 64NE | chr19 | 52212718 | C>A | 14291 | 1 | 0.007 | NO |
| 70NE | chr19 | 52212717 | C>A | 58435 | 4 | 0.007 | NO |
| 55NE | chr19 | 52212730 | G>T | 46529 | 3 | 0.006 | NO |
| 36NE | chr19 | 52212717 | C>A | 46666 | 3 | 0.006 | NO |
| 47NE | chr19 | 52212717 | C>T | 15608 | 1 | 0.006 | NO |
| 47NE | chr19 | 52212718 | C>A | 15610 | 1 | 0.006 | NO |
| 79NE | chr19 | 52212718 | C>A | 62675 | 4 | 0.006 | NO |
| 27NE | chr19 | 52212717 | C>T | 31551 | 2 | 0.006 | NO |
| 54NE | chr19 | 52212727 | G>A | 16032 | 1 | 0.006 | NO |
| 54NE | chr19 | 52212718 | C>G | 16149 | 1 | 0.006 | NO |
| 23NE | chr19 | 52212718 | C>A | 64721 | 4 | 0.006 | NO |
| 59NE | chr19 | 52212730 | G>A | 50269 | 3 | 0.006 | NO |
| 53NE | chr19 | 52212717 | C>T | 17358 | 1 | 0.006 | NO |
| 53NE | chr19 | 52212718 | C>A | 17369 | 1 | 0.006 | NO |
| 58NE | chr19 | 52212717 | C>A | 53166 | 3 | 0.006 | NO |
| 34NE | chr19 | 52212717 | C>A | 35874 | 2 | 0.006 | NO |
| 35NE | chr19 | 52212730 | G>A | 18147 | 1 | 0.006 | NO |
| 60NE | chr19 | 52212717 | C>G | 18237 | 1 | 0.005 | NO |
| 35NE | chr19 | 52212717 | C>T | 18279 | 1 | 0.005 | NO |
| 35NE | chr19 | 52212718 | C>A | 18300 | 1 | 0.005 | NO |
| 84NE | chr19 | 52212730 | G>A | 19740 | 1 | 0.005 | NO |
| 90NE | chr19 | 52212717 | C>G | 19771 | 1 | 0.005 | NO |
| 84NE | chr19 | 52212717 | C>T | 19982 | 1 | 0.005 | NO |
| 84NE | chr19 | 52212718 | C>G | 19992 | 1 | 0.005 | NO |
| 30NE | chr19 | 52212717 | C>A | 20034 | 1 | 0.005 | NO |
| 19NE | chr19 | 52212717 | C>A | 63908 | 3 | 0.005 | NO |
| 56NE | chr19 | 52212717 | C>A | 21952 | 1 | 0.005 | NO |
| 52NE | chr19 | 52212726 | C>G | 22187 | 1 | 0.005 | NO |
| 52NE | chr19 | 52212718 | C>A | 22298 | 1 | 0.004 | NO |
| 73NE | chr19 | 52212717 | C>A | 67280 | 3 | 0.004 | NO |
| 81NE | chr19 | 52212730 | G>A | 46151 | 2 | 0.004 | NO |
| 36NE | chr19 | 52212718 | C>A | 46709 | 2 | 0.004 | NO |
| 55NE | chr19 | 52212718 | C>T | 48811 | 2 | 0.004 | NO |
| 74NE | chr19 | 52212717 | C>G | 49168 | 2 | 0.004 | NO |
| 28NE | chr19 | 52212717 | C>A | 25543 | 1 | 0.004 | NO |
| 59NE | chr19 | 52212717 | C>A | 52651 | 2 | 0.004 | NO |
| 24NE | chr19 | 52212717 | C>A | 30562 | 1 | 0.003 | NO |
| 55NE | chr19 | 52212727 | G>A | 47458 | 1 | 0.002 | NO |
| 81NE | chr19 | 52212717 | C>A | 48354 | 1 | 0.002 | NO |
| 68NE | chr19 | 52212717 | C>A | 59413 | 1 | 0.002 | NO |
| 01NE | chr19 | 52212717 | C>_ | 3627 | 0 | 0.000 | NO |
| 01NE | chr19 | 52212726 | C>_ | 3553 | 0 | 0.000 | NO |
| 01NE | chr19 | 52212727 | G>_ | 3544 | 0 | 0.000 | NO |
| 02NE | chr19 | 52212717 | C>_ | 4181 | 0 | 0.000 | NO |
| 02NE | chr19 | 52212718 | C>_ | 4188 | 0 | 0.000 | NO |
| 02NE | chr19 | 52212727 | G>_ | 4106 | 0 | 0.000 | NO |
| 03NE | chr19 | 52212730 | G>_ | 4036 | 0 | 0.000 | NO |
| 04NE | chr19 | 52212718 | C>_ | 3733 | 0 | 0.000 | NO |
| 04NE | chr19 | 52212726 | C>_ | 3536 | 0 | 0.000 | NO |
| 05NE | chr19 | 52212717 | C>_ | 3615 | 0 | 0.000 | NO |
| 05NE | chr19 | 52212718 | C>_ | 3628 | 0 | 0.000 | NO |
| 05NE | chr19 | 52212729 | C>_ | 3403 | 0 | 0.000 | NO |
| 05NE | chr19 | 52212730 | G>_ | 3349 | 0 | 0.000 | NO |
| 06NE | chr19 | 52212717 | C>_ | 6141 | 0 | 0.000 | NO |
| 06NE | chr19 | 52212726 | C>_ | 6042 | 0 | 0.000 | NO |
| 06NE | chr19 | 52212727 | G>_ | 6004 | 0 | 0.000 | NO |
| 06NE | chr19 | 52212729 | C>_ | 6029 | 0 | 0.000 | NO |
| 07NE | chr19 | 52212729 | C>_ | 4261 | 0 | 0.000 | NO |
| 08NE | chr19 | 52212718 | C>_ | 5827 | 0 | 0.000 | NO |
| 08NE | chr19 | 52212726 | C>_ | 5737 | 0 | 0.000 | NO |
| 08NE | chr19 | 52212730 | G>_ | 5756 | 0 | 0.000 | NO |
| 09NE | chr19 | 52212717 | C>_ | 5750 | 0 | 0.000 | NO |
| 09NE | chr19 | 52212718 | C>_ | 5766 | 0 | 0.000 | NO |
| 09NE | chr19 | 52212727 | G>_ | 5622 | 0 | 0.000 | NO |
| 10NE | chr19 | 52212717 | C>_ | 5101 | 0 | 0.000 | NO |
| 10NE | chr19 | 52212718 | C>_ | 5091 | 0 | 0.000 | NO |
| 10NE | chr19 | 52212726 | C>_ | 5018 | 0 | 0.000 | NO |
| 10NE | chr19 | 52212729 | C>_ | 5012 | 0 | 0.000 | NO |
| 11NE | chr19 | 52212717 | C>_ | 5462 | 0 | 0.000 | NO |
| 12NE | chr19 | 52212717 | C>_ | 3192 | 0 | 0.000 | NO |
| 12NE | chr19 | 52212718 | C>_ | 3202 | 0 | 0.000 | NO |
| 12NE | chr19 | 52212727 | G>_ | 3150 | 0 | 0.000 | NO |
| 12NE | chr19 | 52212729 | C>_ | 3143 | 0 | 0.000 | NO |
| 13NE | chr19 | 52212718 | C>_ | 6650 | 0 | 0.000 | NO |
| 13NE | chr19 | 52212727 | G>_ | 6504 | 0 | 0.000 | NO |
| 15NE | chr19 | 52212718 | C>_ | 5854 | 0 | 0.000 | NO |
| 17NE | chr19 | 52212730 | G>_ | 3295 | 0 | 0.000 | NO |
| 18NE | chr19 | 52212718 | C>_ | 6843 | 0 | 0.000 | NO |
| 20NE | chr19 | 52212717 | C>_ | 5593 | 0 | 0.000 | NO |
| 21NE | chr19 | 52212717 | C>_ | 6916 | 0 | 0.000 | NO |
| 21NE | chr19 | 52212718 | C>_ | 6923 | 0 | 0.000 | NO |
| 22NE | chr19 | 52212717 | C>_ | 4965 | 0 | 0.000 | NO |
| 22NE | chr19 | 52212718 | C>_ | 4958 | 0 | 0.000 | NO |
| 25NE | chr19 | 52212717 | C>_ | 22101 | 0 | 0.000 | NO |
| 33NE | chr19 | 52212717 | C>_ | 13703 | 0 | 0.000 | NO |
| 38NE | chr19 | 52212717 | C>_ | 9874 | 0 | 0.000 | NO |
| 39NE | chr19 | 52212727 | G>_ | 7868 | 0 | 0.000 | NO |
| 40NE | chr19 | 52212717 | C>_ | 13139 | 0 | 0.000 | NO |
| 41NE | chr19 | 52212718 | C>_ | 7330 | 0 | 0.000 | NO |
| 41NE | chr19 | 52212730 | G>_ | 7292 | 0 | 0.000 | NO |
| 43NE | chr19 | 52212717 | C>_ | 11125 | 0 | 0.000 | NO |
| 43NE | chr19 | 52212727 | G>_ | 10953 | 0 | 0.000 | NO |
| 44NE | chr19 | 52212718 | C>_ | 8781 | 0 | 0.000 | NO |
| 44NE | chr19 | 52212726 | C>_ | 8620 | 0 | 0.000 | NO |
| 44NE | chr19 | 52212727 | G>_ | 8654 | 0 | 0.000 | NO |
| 48NE | chr19 | 52212717 | C>_ | 8759 | 0 | 0.000 | NO |
| 48NE | chr19 | 52212727 | G>_ | 8715 | 0 | 0.000 | NO |
| 48NE | chr19 | 52212730 | G>_ | 8687 | 0 | 0.000 | NO |
| 49NE | chr19 | 52212717 | C>_ | 11359 | 0 | 0.000 | NO |
| 54NE | chr19 | 52212717 | C>_ | 16133 | 0 | 0.000 | NO |
| 62NE | chr19 | 52212718 | C>_ | 4082 | 0 | 0.000 | NO |
| 62NE | chr19 | 52212730 | G>_ | 4041 | 0 | 0.000 | NO |
| 65NE | chr19 | 52212717 | C>_ | 3452 | 0 | 0.000 | NO |
| 65NE | chr19 | 52212726 | C>_ | 3423 | 0 | 0.000 | NO |
| 65NE | chr19 | 52212727 | G>_ | 3403 | 0 | 0.000 | NO |
| 65NE | chr19 | 52212730 | G>_ | 3433 | 0 | 0.000 | NO |
| 66NE | chr19 | 52212717 | C>_ | 5549 | 0 | 0.000 | NO |
| 66NE | chr19 | 52212729 | C>_ | 5512 | 0 | 0.000 | NO |
| 66NE | chr19 | 52212730 | G>_ | 5440 | 0 | 0.000 | NO |
| 67NE | chr19 | 52212717 | C>_ | 10757 | 0 | 0.000 | NO |
| 75NE | chr19 | 52212717 | C>_ | 6979 | 0 | 0.000 | NO |
| 75NE | chr19 | 52212730 | G>_ | 6885 | 0 | 0.000 | NO |
| 76NE | chr19 | 52212729 | C>_ | 11039 | 0 | 0.000 | NO |
| 78NE | chr19 | 52212717 | C>_ | 3150 | 0 | 0.000 | NO |
| 78NE | chr19 | 52212718 | C>_ | 3151 | 0 | 0.000 | NO |
| 78NE | chr19 | 52212726 | C>_ | 3142 | 0 | 0.000 | NO |
| 78NE | chr19 | 52212730 | G>_ | 3142 | 0 | 0.000 | NO |
| 82NE | chr19 | 52212717 | C>_ | 6877 | 0 | 0.000 | NO |
| 85NE | chr19 | 52212717 | C>_ | 2375 | 0 | 0.000 | NO |
| 85NE | chr19 | 52212718 | C>_ | 2385 | 0 | 0.000 | NO |
| 85NE | chr19 | 52212726 | C>_ | 2339 | 0 | 0.000 | NO |
| 85NE | chr19 | 52212727 | G>_ | 2338 | 0 | 0.000 | NO |
| 85NE | chr19 | 52212729 | C>_ | 2345 | 0 | 0.000 | NO |
| 85NE | chr19 | 52212730 | G>_ | 2338 | 0 | 0.000 | NO |
| 86NE | chr19 | 52212718 | C>_ | 2979 | 0 | 0.000 | NO |
| 86NE | chr19 | 52212726 | C>_ | 2916 | 0 | 0.000 | NO |
| 87NE | chr19 | 52212717 | C>_ | 5612 | 0 | 0.000 | NO |
| 87NE | chr19 | 52212729 | C>_ | 5496 | 0 | 0.000 | NO |
| 88NE | chr19 | 52212717 | C>_ | 5967 | 0 | 0.000 | NO |
| 88NE | chr19 | 52212718 | C>_ | 5975 | 0 | 0.000 | NO |
| 88NE | chr19 | 52212730 | G>_ | 5887 | 0 | 0.000 | NO |
| 89NE | chr19 | 52212718 | C>_ | 9626 | 0 | 0.000 | NO |
| 92NE | chr19 | 52212717 | C>_ | 944 | 0 | 0.000 | NO |
| 92NE | chr19 | 52212718 | C>_ | 944 | 0 | 0.000 | NO |
| 92NE | chr19 | 52212726 | C>_ | 940 | 0 | 0.000 | NO |
| 92NE | chr19 | 52212727 | G>_ | 939 | 0 | 0.000 | NO |
| 92NE | chr19 | 52212729 | C>_ | 940 | 0 | 0.000 | NO |
| 92NE | chr19 | 52212730 | G>_ | 938 | 0 | 0.000 | NO |
| 93NE | chr19 | 52212717 | C>_ | 1700 | 0 | 0.000 | NO |
| 93NE | chr19 | 52212718 | C>_ | 1700 | 0 | 0.000 | NO |
| 93NE | chr19 | 52212726 | C>_ | 1687 | 0 | 0.000 | NO |
| 93NE | chr19 | 52212730 | G>_ | 1677 | 0 | 0.000 | NO |
| 98NE | chr19 | 52212717 | C>_ | 2440 | 0 | 0.000 | NO |
| 98NE | chr19 | 52212718 | C>_ | 2444 | 0 | 0.000 | NO |
| 98NE | chr19 | 52212726 | C>_ | 2428 | 0 | 0.000 | NO |
| 98NE | chr19 | 52212729 | C>_ | 2428 | 0 | 0.000 | NO |
| ^1^Somatic mutation, somatic mutation calls were described in Supplementary method. | | | | | | | |

| **Table S7. Determination of threshold for somatic *KRAS* mutations.** | | | | | |
| --- | --- | --- | --- | --- | --- |
| Sample_ID | VAF (%) | | | | |
|  | *25245347 | *25245348 | *25245349 | *25245350 | *25245351 |
| 01NM | 0.000 | 0.013 | 0.008 | 0.156 | 0.013 |
| 02NM | 0.006 | 0.006 | 0.006 | 0.006 | 0.026 |
| 03NM | 0.017 | 0.000 | 0.000 | 0.000 | 0.000 |
| 04NM | 0.000 | 0.000 | 0.041 | 0.000 | 0.021 |
| 05NM | 0.006 | 0.006 | 0.018 | 0.006 | 0.024 |
| 06NM | 0.006 | 0.006 | 0.006 | 0.011 | 0.029 |
| 07NM | 0.006 | 0.006 | 0.023 | 0.017 | 0.040 |
| 08NM | 0.000 | 0.000 | 0.007 | 0.000 | 0.015 |
| 09NM | 0.008 | 0.008 | 0.008 | 0.000 | 0.008 |
| 10NM | 0.005 | 0.005 | 0.015 | 0.010 | 0.015 |
| 11NM | 0.000 | 0.000 | 0.000 | 0.000 | 0.025 |
| 12NM | 0.023 | 0.046 | 0.000 | 0.023 | 0.046 |
| 13NM | 0.010 | 0.010 | 0.010 | 0.010 | 0.010 |
| 14NM | 0.011 | 0.011 | 0.015 | 0.007 | 0.007 |
| 15NM | 0.020 | 0.011 | 0.015 | 0.009 | 0.022 |
| 16NM | 0.010 | 0.010 | 0.014 | 0.007 | 0.017 |
| 17NM | 0.013 | 0.010 | 0.013 | 0.010 | 0.013 |
| 18NM | 0.013 | 0.009 | 0.016 | 0.016 | 0.010 |
| 19NM | 0.012 | 0.007 | 0.014 | 0.000 | 0.010 |
| 20NM | 0.011 | 0.013 | 0.018 | 0.008 | 0.016 |
| 21NM | 0.010 | 0.012 | 0.012 | 0.008 | 0.008 |
| 22NM | 0.003 | 0.016 | 0.059 | 0.008 | 0.016 |
| 23NM | 0.011 | 0.005 | 0.005 | 0.008 | 0.013 |
| 24NM | 0.014 | 0.014 | 0.014 | 0.000 | 0.000 |
| 25NM | 0.000 | 0.013 | 0.013 | 0.000 | 0.013 |
| 26NM | 0.013 | 0.013 | 0.025 | 0.141 | 0.013 |
| 27NM | 0.000 | 0.027 | 0.018 | 0.152 | 0.009 |
| 28NM | 0.000 | 0.000 | 0.020 | 0.100 | 0.000 |
| 29NM | 0.000 | 0.007 | 0.014 | 0.007 | 0.014 |
| 30NM | 0.036 | 0.036 | 0.000 | 0.073 | 0.036 |
| 31NM | 0.000 | 0.008 | 0.023 | 0.122 | 0.008 |
| 32NM | 0.000 | 0.042 | 0.014 | 0.156 | 0.028 |
| 33NM | 0.000 | 0.011 | 0.011 | 0.125 | 0.011 |
| 34NM | 0.027 | 0.007 | 0.020 | 0.149 | 0.020 |
| 35NM | 0.000 | 0.022 | 0.000 | 0.000 | 0.022 |
| 36NM | 0.006 | 0.006 | 0.018 | 0.501 | 0.006 |
| 37NM | 0.000 | 0.019 | 0.019 | 0.000 | 0.019 |
| 38NM | 0.000 | 0.000 | 0.016 | 0.000 | 0.000 |
| 39NM | 0.000 | 0.011 | 0.011 | 0.296 | 0.000 |
| 40NM | 0.016 | 0.016 | 0.000 | 0.032 | 0.000 |
| 41NM | 0.012 | 0.012 | 0.012 | 0.012 | 0.000 |
| 42NM | 0.000 | 0.028 | 0.000 | 0.000 | 0.000 |
| 43NM | 0.008 | 0.016 | 0.016 | 0.008 | 0.008 |
| 44NM | 0.000 | 0.007 | 0.007 | 0.000 | 0.007 |
| 45NM | 0.010 | 0.000 | 0.010 | 0.000 | 0.010 |
| 46NM | 0.007 | 0.014 | 0.014 | 0.007 | 0.007 |
| 47NM | 0.012 | 0.006 | 0.012 | 0.012 | 0.029 |
| 48NM | 0.007 | 0.007 | 0.022 | 0.007 | 0.014 |
| 49NM | 0.005 | 0.025 | 0.015 | 0.005 | 0.020 |
| 50NM | 0.038 | 0.000 | 0.077 | 0.117 | 0.039 |
| 51NM | 0.000 | 0.000 | 0.022 | 0.043 | 0.022 |
| 52NM | 0.003 | 0.009 | 0.009 | 0.006 | 0.009 |
| 53NM | 0.009 | 0.009 | 0.013 | 0.008 | 0.009 |
| 54NM | 0.000 | 0.004 | 0.004 | 0.004 | 0.013 |
| 55NM | 0.000 | 0.006 | 0.010 | 0.003 | 0.000 |
| 57NM | 0.006 | 0.009 | 0.009 | 0.009 | 0.006 |
| 58NM | 0.005 | 0.005 | 0.010 | 0.000 | 0.159 |
| 59NM | 0.010 | 0.015 | 0.010 | 0.010 | 0.010 |
| 60NM | 0.000 | 0.006 | 0.006 | 0.019 | 0.025 |
| 61NM | 0.009 | 0.005 | 0.032 | 0.087 | 0.009 |
| 62NM | 0.000 | 0.000 | 0.032 | 0.032 | 0.064 |
| 63NM | 0.012 | 0.006 | 0.018 | 0.012 | 0.012 |
| 64NM | 0.010 | 0.010 | 0.020 | 0.010 | 0.031 |
| 65NM | 0.000 | 0.008 | 0.012 | 0.004 | 0.020 |
| 56NM | 0.000 | 0.017 | 0.017 | 0.069 | 0.017 |
| 66NM | 0.000 | 0.032 | 0.032 | 0.097 | 0.000 |
| 67NM | 0.000 | 0.023 | 0.023 | 0.069 | 0.023 |
| 68NM | 0.000 | 0.007 | 0.007 | 0.007 | 0.000 |
| 69NM | 0.009 | 0.009 | 0.027 | 0.009 | 0.000 |
| 70NM | 0.000 | 0.000 | 0.014 | 0.000 | 0.000 |
| 71NM | 0.015 | 0.000 | 0.015 | 0.030 | 0.015 |
| 72NM | 0.022 | 0.011 | 0.011 | 0.011 | 0.022 |
| 73NM | 0.000 | 0.005 | 0.009 | 0.005 | 0.005 |
| 74NM | 0.007 | 0.014 | 0.014 | 0.014 | 0.000 |
| 75NM | 0.014 | 0.027 | 0.013 | 0.013 | 0.000 |
| 76NM | 0.009 | 0.018 | 0.000 | 0.009 | 0.009 |
| 77NM | 0.094 | 0.073 | 0.021 | 0.104 | 0.104 |
| 78NM | 0.000 | 0.014 | 0.014 | 0.000 | 0.014 |
| 79NM | 0.018 | 0.006 | 0.006 | 0.012 | 0.006 |
| 80NM | 0.010 | 0.004 | 0.010 | 0.042 | 0.010 |
| 81NM | 0.008 | 0.003 | 0.015 | 0.004 | 0.007 |
| 82NM | 0.000 | 0.000 | 0.007 | 0.013 | 0.013 |
| 83NM | 0.000 | 0.035 | 0.017 | 0.122 | 0.000 |
| 84NM | 0.011 | 0.011 | 0.033 | 0.000 | 0.000 |
| 85NM | 0.000 | 0.023 | 0.000 | 0.000 | 0.000 |
| 86NM | 0.000 | 0.000 | 0.000 | 0.000 | 0.070 |
| 87NM | 0.006 | 0.007 | 0.013 | 0.007 | 0.024 |
| 88NM | 0.000 | 0.012 | 0.012 | 0.012 | 0.024 |
| 89NM | 0.000 | 0.013 | 0.027 | 0.013 | 0.020 |
| 90NM | 0.005 | 0.005 | 0.022 | 0.016 | 0.011 |
| 91NM | 0.005 | 0.005 | 0.015 | 0.015 | 0.025 |
| 92NM | 0.006 | 0.006 | 0.013 | 0.006 | 0.013 |
| 93NM | 0.000 | 0.011 | 0.032 | 0.011 | 0.011 |
| 94NM | 0.008 | 0.016 | 0.008 | 0.008 | 0.008 |
| 95NM | 0.059 | 0.000 | 0.000 | 0.029 | 0.000 |
| 96NM | 0.000 | 0.010 | 0.021 | 0.005 | 0.010 |
| 97NM | 0.008 | 0.008 | 0.008 | 0.016 | 0.000 |
| 98NM | 0.006 | 0.013 | 0.006 | 0.013 | 0.586 |
|  |  |  |  |  |  |
| Mean | 0.008 | 0.011 | 0.015 | 0.034 | 0.022 |
| SD | 0.013 | 0.011 | 0.011 | 0.069 | 0.061 |
| Mean+3SD | 0.046 | 0.045 | 0.049 | **0.241** | 0.206 |
| Threshhold of somatic *KRAS* mutation: 0.241 % | | | |  |  |
| *hg38: *KRAS* p.G12-G13 locus (chr12: 25245347-25245351) | | | | |  |

| **Table S8. Determination of threshold for somatic *PIK3CA* mutations.** | | | |
| --- | --- | --- | --- |
| Sample_ID | VAF (%) | | |
|  | *179234296 | *179234297 | *179234298 |
| 01NM | 0.042 | 0.023 | 0.008 |
| 02NM | 0.052 | 0.023 | 0.005 |
| 03NM | 0.037 | 0.026 | 0.007 |
| 04NM | 0.049 | 0.025 | 0.009 |
| 05NM | 0.053 | 0.030 | 0.009 |
| 06NM | 0.045 | 0.028 | 0.008 |
| 07NM | 0.040 | 0.029 | 0.010 |
| 08NM | 0.051 | 0.030 | 0.001 |
| 09NM | 0.043 | 0.024 | 0.002 |
| 10NM | 0.049 | 0.024 | 0.007 |
| 11NM | 0.057 | 0.015 | 0.005 |
| 12NM | 0.039 | 0.016 | 0.007 |
| 13NM | 0.012 | 0.028 | 0.029 |
| 14NM | 0.010 | 0.013 | 0.012 |
| 15NM | 0.060 | 0.015 | 0.013 |
| 16NM | 0.007 | 0.013 | 0.018 |
| 17NM | 0.018 | 0.014 | 0.015 |
| 18NM | 0.014 | 0.013 | 0.021 |
| 19NM | 0.014 | 0.038 | 0.031 |
| 20NM | 0.038 | 0.235 | 0.016 |
| 21NM | 0.017 | 0.017 | 0.016 |
| 22NM | 0.013 | 0.020 | 0.026 |
| 23NM | 0.092 | 0.015 | 0.023 |
| 24NM | 0.000 | 0.015 | 0.037 |
| 25NM | 0.012 | 0.008 | 0.008 |
| 26NM | 0.020 | 0.073 | 0.000 |
| 27NM | 0.018 | 0.045 | 0.005 |
| 28NM | 0.013 | 0.052 | 0.000 |
| 29NM | 0.008 | 0.016 | 0.005 |
| 30NM | 0.007 | 0.037 | 0.007 |
| 31NM | 0.013 | 0.065 | 0.022 |
| 32NM | 0.006 | 0.054 | 0.024 |
| 33NM | 0.013 | 0.063 | 0.004 |
| 34NM | 0.000 | 0.302 | 0.094 |
| 35NM | 0.002 | 0.005 | 0.018 |
| 36NM | 0.007 | 0.005 | 0.005 |
| 37NM | 0.013 | 0.006 | 0.016 |
| 38NM | 0.013 | 0.012 | 0.013 |
| 39NM | 0.022 | 0.007 | 0.011 |
| 40NM | 0.012 | 0.004 | 0.016 |
| 41NM | 0.011 | 0.000 | 0.025 |
| 42NM | 0.015 | 0.015 | 0.008 |
| 43NM | 0.008 | 0.021 | 0.005 |
| 44NM | 0.012 | 0.012 | 0.021 |
| 45NM | 0.009 | 0.000 | 0.009 |
| 46NM | 0.013 | 0.013 | 0.009 |
| 47NM | 0.003 | 0.003 | 0.019 |
| 48NM | 0.009 | 0.004 | 0.000 |
| 49NM | 0.042 | 0.022 | 0.012 |
| 50NM | 0.021 | 0.048 | 0.014 |
| 51NM | 0.009 | 0.006 | 0.012 |
| 52NM | 0.014 | 0.005 | 0.009 |
| 53NM | 0.007 | 0.008 | 0.012 |
| 54NM | 0.006 | 0.005 | 0.008 |
| 55NM | 0.007 | 0.008 | 0.007 |
| 57NM | 0.007 | 0.028 | 0.006 |
| 58NM | 0.008 | 0.004 | 0.009 |
| 59NM | 0.011 | 0.005 | 0.006 |
| 60NM | 0.052 | 0.032 | 0.005 |
| 61NM | 0.047 | 0.026 | 0.004 |
| 62NM | 0.038 | 0.023 | 0.003 |
| 63NM | 0.057 | 0.021 | 0.004 |
| 64NM | 0.035 | 0.029 | 0.007 |
| 65NM | 0.048 | 0.028 | 0.003 |
| 56NM | 0.011 | 0.028 | 0.011 |
| 66NM | 0.024 | 0.045 | 0.000 |
| 67NM | 0.025 | 0.025 | 0.025 |
| 68NM | 0.002 | 0.005 | 0.010 |
| 69NM | 0.002 | 0.023 | 0.005 |
| 70NM | 0.012 | 0.009 | 0.006 |
| 71NM | 0.020 | 0.005 | 0.005 |
| 72NM | 0.011 | 0.011 | 0.011 |
| 73NM | 0.006 | 0.017 | 0.000 |
| 74NM | 0.006 | 0.011 | 0.006 |
| 75NM | 0.005 | 0.005 | 0.015 |
| 76NM | 0.009 | 0.005 | 0.009 |
| 77NM | 0.004 | 0.009 | 0.015 |
| 78NM | 0.004 | 0.102 | 0.002 |
| 79NM | 0.004 | 0.008 | 0.008 |
| 80NM | 0.008 | 0.007 | 0.005 |
| 81NM | 0.010 | 0.005 | 0.007 |
| 82NM | 0.058 | 0.017 | 0.008 |
| 83NM | 0.013 | 0.066 | 0.011 |
| 84NM | 0.008 | 0.013 | 0.022 |
| 85NM | 0.049 | 0.030 | 0.005 |
| 86NM | 0.044 | 0.020 | 0.004 |
| 87NM | 0.040 | 0.029 | 0.007 |
| 88NM | 0.031 | 0.032 | 0.017 |
| 89NM | 0.013 | 0.013 | 0.018 |
| 90NM | 0.007 | 0.004 | 0.019 |
| 91NM | 0.014 | 0.006 | 0.020 |
| 92NM | 0.077 | 0.009 | 0.015 |
| 93NM | 0.007 | 0.007 | 0.014 |
| 94NM | 0.004 | 0.004 | 0.012 |
| 95NM | 0.011 | 0.009 | 0.025 |
| 96NM | 0.016 | 0.011 | 0.020 |
| 97NM | 0.009 | 0.011 | 0.013 |
| 98NM | 0.007 | 0.081 | 0.016 |
|  |  |  |  |
| Mean | 0.021 | 0.026 | 0.012 |
| SD | 0.019 | 0.040 | 0.011 |
| Mean+3SD | 0.079 | **0.146** | 0.046 |
| Threshold of somatic *PIK3CA* mutation: 0.146 % | | | |
| *hg38: *PIK3CA* p.H1047 locus (chr3: 179234296-179234298) | | | |

| **Table S9. Determination of threshold for somatic *PPP2R1A* mutations.** | | | | | | | |  |
| --- | --- | --- | --- | --- | --- | --- | --- | --- |
| Sample_ID | VAF (%) | | | | | | | |
|  | *52212717 | *52212718 | *52212726 | *52212727 | *52212728 | *52212729 | *52212730 | *52212731 |
| 01NM | 0.020 | 0.000 | 0.060 | 0.020 | 0.000 | 0.041 | 0.000 | 0.021 |
| 02NM | 0.000 | 0.000 | 0.000 | 0.000 | 0.000 | 0.056 | 0.028 | 0.000 |
| 03NM | 0.000 | 0.048 | 0.000 | 0.000 | 0.000 | 0.049 | 0.049 | 0.000 |
| 04NM | 0.000 | 0.000 | 0.021 | 0.021 | 0.000 | 0.021 | 0.021 | 0.022 |
| 05NM | 0.000 | 0.000 | 0.048 | 0.000 | 0.000 | 0.049 | 0.000 | 0.000 |
| 06NM | 0.031 | 0.031 | 0.032 | 0.032 | 0.032 | 0.032 | 0.000 | 0.000 |
| 07NM | 0.000 | 0.000 | 0.015 | 0.015 | 0.015 | 0.031 | 0.137 | 0.016 |
| 08NM | 0.000 | 0.019 | 0.038 | 0.000 | 0.000 | 0.019 | 0.019 | 0.020 |
| 09NM | 0.000 | 0.000 | 0.000 | 0.023 | 0.000 | 0.000 | 0.000 | 0.023 |
| 10NM | 0.000 | 0.015 | 0.000 | 0.015 | 0.015 | 0.046 | 0.030 | 0.000 |
| 11NM | 0.000 | 0.000 | 0.086 | 0.014 | 0.014 | 0.014 | 0.000 | 0.029 |
| 12NM | 0.000 | 0.000 | 0.040 | 0.041 | 0.000 | 0.000 | 0.081 | 0.000 |
| 13NM | 0.012 | 0.010 | 0.036 | 0.019 | 0.019 | 0.027 | 0.022 | 0.020 |
| 14NM | 0.010 | 0.009 | 0.022 | 0.038 | 0.017 | 0.022 | 0.034 | 0.015 |
| 15NM | 0.000 | 0.041 | 0.041 | 0.325 | 0.000 | 0.244 | 0.041 | 0.041 |
| 16NM | 0.012 | 0.012 | 0.023 | 0.025 | 0.013 | 0.011 | 0.018 | 0.011 |
| 17NM | 0.014 | 0.014 | 0.022 | 0.024 | 0.012 | 0.033 | 0.020 | 0.018 |
| 18NM | 0.016 | 0.016 | 0.016 | 0.034 | 0.019 | 0.028 | 0.053 | 0.022 |
| 19NM | 0.007 | 0.011 | 0.032 | 0.062 | 0.032 | 0.024 | 0.015 | 0.006 |
| 20NM | 0.016 | 0.009 | 0.059 | 0.027 | 0.016 | 0.032 | 0.039 | 0.030 |
| 21NM | 0.000 | 0.000 | 0.000 | 0.000 | 0.000 | 0.000 | 0.082 | 0.000 |
| 22NM | 0.005 | 0.008 | 0.025 | 0.020 | 0.010 | 0.021 | 0.018 | 0.016 |
| 23NM | 0.000 | 0.000 | 0.104 | 0.103 | 0.000 | 0.103 | 0.000 | 0.000 |
| 24NM | 0.010 | 0.010 | 0.040 | 0.025 | 0.010 | 0.005 | 0.035 | 0.020 |
| 25NM | 0.011 | 0.005 | 0.022 | 0.022 | 0.011 | 0.033 | 0.016 | 0.011 |
| 26NM | 0.000 | 0.026 | 0.031 | 0.016 | 0.010 | 0.057 | 0.016 | 0.042 |
| 27NM | 0.004 | 0.030 | 0.034 | 0.021 | 0.017 | 0.051 | 0.021 | 0.021 |
| 28NM | 0.000 | 0.066 | 0.026 | 0.040 | 0.000 | 0.040 | 0.013 | 0.013 |
| 29NM | 0.010 | 0.010 | 0.030 | 0.007 | 0.003 | 0.050 | 0.034 | 0.014 |
| 30NM | 0.005 | 0.016 | 0.048 | 0.048 | 0.011 | 0.026 | 0.005 | 0.016 |
| 31NM | 0.010 | 0.025 | 0.010 | 0.030 | 0.025 | 0.040 | 0.020 | 0.030 |
| 32NM | 0.008 | 0.031 | 0.008 | 0.016 | 0.008 | 0.047 | 0.016 | 0.008 |
| 33NM | 0.000 | 0.039 | 0.026 | 0.017 | 0.026 | 0.017 | 0.018 | 0.013 |
| 34NM | 0.007 | 0.007 | 0.014 | 0.016 | 0.009 | 0.023 | 0.009 | 0.012 |
| 35NM | 0.000 | 0.021 | 0.021 | 0.000 | 0.042 | 0.063 | 0.000 | 0.021 |
| 36NM | 0.010 | 0.005 | 0.011 | 0.016 | 0.000 | 0.074 | 0.016 | 0.000 |
| 37NM | 0.006 | 0.003 | 0.028 | 0.003 | 0.006 | 0.083 | 0.009 | 0.003 |
| 38NM | 0.008 | 0.008 | 0.024 | 0.016 | 0.008 | 0.022 | 0.014 | 0.014 |
| 39NM | 0.003 | 0.008 | 0.016 | 0.016 | 0.005 | 0.026 | 0.097 | 0.005 |
| 40NM | 0.006 | 0.003 | 0.014 | 0.003 | 0.012 | 0.037 | 0.006 | 0.023 |
| 41NM | 0.010 | 0.005 | 0.021 | 0.029 | 0.011 | 0.018 | 0.013 | 0.016 |
| 42NM | 0.000 | 0.012 | 0.009 | 0.025 | 0.003 | 0.029 | 0.013 | 0.010 |
| 43NM | 0.006 | 0.012 | 0.030 | 0.009 | 0.009 | 0.030 | 0.009 | 0.012 |
| 44NM | 0.007 | 0.007 | 0.068 | 0.009 | 0.007 | 0.016 | 0.016 | 0.007 |
| 45NM | 0.010 | 0.010 | 0.033 | 0.052 | 0.007 | 0.023 | 0.013 | 0.010 |
| 46NM | 0.011 | 0.005 | 0.024 | 0.024 | 0.011 | 0.019 | 0.008 | 0.005 |
| 47NM | 0.004 | 0.010 | 0.042 | 0.021 | 0.014 | 0.048 | 0.012 | 0.066 |
| 48NM | 0.005 | 0.005 | 0.014 | 0.095 | 0.010 | 0.017 | 0.012 | 0.007 |
| 49NM | 0.009 | 0.007 | 0.130 | 0.014 | 0.005 | 0.021 | 0.019 | 0.012 |
| 50NM | 0.011 | 0.016 | 0.022 | 0.027 | 0.005 | 0.055 | 0.055 | 0.033 |
| 51NM | 0.000 | 0.009 | 0.009 | 0.009 | 0.020 | 0.019 | 0.038 | 0.000 |
| 52NM | 0.006 | 0.007 | 0.017 | 0.015 | 0.106 | 0.031 | 0.006 | 0.008 |
| 53NM | 0.007 | 0.011 | 0.017 | 0.013 | 0.050 | 0.027 | 0.136 | 0.006 |
| 54NM | 0.003 | 0.011 | 0.014 | 0.008 | 0.068 | 0.099 | 0.008 | 0.006 |
| 55NM | 0.014 | 0.012 | 0.041 | 0.011 | 0.060 | 0.027 | 0.007 | 0.007 |
| 57NM | 0.006 | 0.013 | 0.014 | 0.012 | 0.085 | 0.023 | 0.045 | 0.005 |
| 58NM | 0.006 | 0.004 | 0.072 | 0.037 | 0.054 | 0.089 | 0.008 | 0.008 |
| 59NM | 0.009 | 0.010 | 0.040 | 0.007 | 0.112 | 0.028 | 0.007 | 0.004 |
| 60NM | 0.005 | 0.005 | 0.022 | 0.038 | 0.017 | 0.038 | 0.011 | 0.011 |
| 61NM | 0.007 | 0.007 | 0.014 | 0.021 | 0.007 | 0.028 | 0.028 | 0.063 |
| 62NM | 0.007 | 0.007 | 0.014 | 0.021 | 0.007 | 0.028 | 0.028 | 0.063 |
| 63NM | 0.006 | 0.000 | 0.012 | 0.024 | 0.012 | 0.018 | 0.024 | 0.030 |
| 64NM | 0.006 | 0.006 | 0.032 | 0.032 | 0.006 | 0.013 | 0.025 | 0.006 |
| 65NM | 0.006 | 0.006 | 0.032 | 0.032 | 0.006 | 0.013 | 0.025 | 0.006 |
| 56NM | 0.006 | 0.028 | 0.061 | 0.017 | 0.000 | 0.017 | 0.006 | 0.022 |
| 66NM | 0.006 | 0.028 | 0.061 | 0.017 | 0.000 | 0.017 | 0.006 | 0.022 |
| 67NM | 0.000 | 0.000 | 0.033 | 0.000 | 0.033 | 0.000 | 0.000 | 0.034 |
| 68NM | 0.000 | 0.022 | 0.034 | 0.022 | 0.034 | 0.022 | 0.023 | 0.022 |
| 69NM | 0.009 | 0.057 | 0.019 | 0.038 | 0.019 | 0.028 | 0.019 | 0.000 |
| 70NM | 0.000 | 0.029 | 0.043 | 0.007 | 0.014 | 0.007 | 0.051 | 0.014 |
| 71NM | 0.000 | 0.074 | 0.000 | 0.074 | 0.000 | 0.000 | 0.074 | 0.000 |
| 72NM | 0.003 | 0.028 | 0.038 | 0.042 | 0.010 | 0.042 | 0.008 | 0.022 |
| 73NM | 0.005 | 0.018 | 0.023 | 0.018 | 0.023 | 0.111 | 0.011 | 0.023 |
| 74NM | 0.005 | 0.016 | 0.049 | 0.015 | 0.007 | 0.084 | 0.013 | 0.024 |
| 75NM | 0.004 | 0.022 | 0.035 | 0.042 | 0.011 | 0.067 | 0.016 | 0.011 |
| 76NM | 0.005 | 0.042 | 0.017 | 0.028 | 0.009 | 0.026 | 0.019 | 0.026 |
| 77NM | 0.002 | 0.022 | 0.032 | 0.016 | 0.011 | 0.032 | 0.013 | 0.021 |
| 78NM | 0.004 | 0.009 | 0.012 | 0.032 | 0.016 | 0.035 | 0.021 | 0.018 |
| 79NM | 0.000 | 0.034 | 0.043 | 0.009 | 0.026 | 0.026 | 0.034 | 0.009 |
| 80NM | 0.000 | 0.033 | 0.028 | 0.019 | 0.019 | 0.009 | 0.010 | 0.019 |
| 81NM | 0.006 | 0.003 | 0.065 | 0.018 | 0.009 | 0.059 | 0.009 | 0.003 |
| 82NM | 0.009 | 0.009 | 0.013 | 0.028 | 0.085 | 0.050 | 0.019 | 0.006 |
| 83NM | 0.010 | 0.007 | 0.018 | 0.038 | 0.091 | 0.013 | 0.018 | 0.012 |
| 84NM | 0.008 | 0.017 | 0.016 | 0.041 | 0.066 | 0.021 | 0.032 | 0.003 |
| 85NM | 0.010 | 0.028 | 0.010 | 0.042 | 0.011 | 0.035 | 0.063 | 0.017 |
| 86NM | 0.008 | 0.031 | 0.023 | 0.023 | 0.016 | 0.023 | 0.032 | 0.016 |
| 87NM | 0.013 | 0.007 | 0.026 | 0.020 | 0.007 | 0.020 | 0.013 | 0.013 |
| 88NM | 0.000 | 0.000 | 0.040 | 0.020 | 0.021 | 0.041 | 0.041 | 0.000 |
| 89NM | 0.000 | 0.000 | 0.040 | 0.020 | 0.021 | 0.041 | 0.041 | 0.000 |
| 90NM | 0.000 | 0.000 | 0.000 | 0.015 | 0.015 | 0.076 | 0.030 | 0.016 |
| 91NM | 0.000 | 0.000 | 0.022 | 0.000 | 0.022 | 0.043 | 0.064 | 0.045 |
| 92NM | 0.018 | 0.000 | 0.037 | 0.000 | 0.037 | 0.019 | 0.037 | 0.019 |
| 93NM | 0.006 | 0.006 | 0.028 | 0.045 | 0.011 | 0.039 | 0.039 | 0.006 |
| 94NM | 0.016 | 0.006 | 0.029 | 0.022 | 0.010 | 0.013 | 0.026 | 0.006 |
| 95NM | 0.007 | 0.000 | 0.007 | 0.051 | 0.015 | 0.029 | 0.007 | 0.022 |
| 96NM | 0.000 | 0.000 | 0.000 | 0.099 | 0.000 | 0.197 | 0.000 | 0.000 |
| 97NM | 0.000 | 0.000 | 0.008 | 0.016 | 0.016 | 0.031 | 0.078 | 0.016 |
| 98NM | 0.013 | 0.007 | 0.017 | 0.013 | 0.017 | 0.024 | 0.044 | 0.015 |
|  |  |  |  |  |  |  |  |  |
| Mean | 0.006 | 0.014 | 0.028 | 0.027 | 0.018 | 0.037 | 0.026 | 0.015 |
| SD | 0.006 | 0.015 | 0.022 | 0.036 | 0.023 | 0.035 | 0.026 | 0.013 |
| Mean+3SD | 0.023 | 0.057 | 0.093 | 0.135 | 0.087 | **0.142** | 0.103 | 0.055 |
| Threshold of somatic *PPP2R1A* mutation: 0.142 % | | | | | |  |  |  |
| ^1^hg38: *PPP2R1A* p.P179/R182-183 locus (chr19: 52212717-52212731) | | | | | | |  |  |

| **Table S10. Relationship between patient characteristics and *KRAS* mutation status in normal endometrium related to Table 1.** | | | | | | | | | |  |  |
| --- | --- | --- | --- | --- | --- | --- | --- | --- | --- | --- | --- |
|  | | | Value (%) | | | | *Statistics | | |  |  |
|  | | | *KRAS* wild type | | *KRAS* mutated | |  |  |  |  |  |
| Characteristic | | | (N=40) | | (N=58) | |  |  |  |  |  |
| Gravidity >1, no. (%) | | | 12/40 (30.00) | | 38/58 (65.52) | | 0.001 | | |  |  |
| 1 | | | 5/40 (12.50) | | 16/58 (27.59) | |  | | |  |  |
| 2 | | | 5/40 (12.50) | | 11/58 (18.97) | |  | | |  |  |
| 3 | | | 1/40 (2.50) | | 8/58 (13.79) | |  | | |  |  |
| 4 | | | 1/40 (2.50) | | 2/58 (3.45) | |  | | |  |  |
| 6 | | | 0/40 (0.00) | | 1/58 (1.72) | |  | | |  |  |
|  | | |  | |  | |  | | |  |  |
| Parity >1, no. (%) | | | 11/40 (27.50) | | 33/58 (56.90) | | 0.007 | | |  |  |
| 1 | | | 5/40 (12.50) | | 15/58 (25.86) | |  | | |  |  |
| 2 | | | 5/40 (12.50) | | 10/58 (17.24) | |  | | |  |  |
| 3 | | | 1/40 (2.50) | | 8/58 (13.79) | |  | | |  |  |
|  | | |  | |  | |  | | |  |  |
|  | | |  | |  | |  | | |  |  |
| Abortion or stillborn >1, no. (%) | | | 3/40 (7.50) | | 11/58 (18.97) | | n.s. | | |  |  |
| 1 | | | 2/40 (5.00) | | 7/58 (12.07) | |  | | |  |  |
| 2 | | | 1/40 (2.50) | | 3/58 (5.17) | |  | | |  |  |
| 3 | | | 0/40 (0.00) | | 1/58 (1.72) | |  | | |  |  |
|  | | |  | |  | |  | | |  |  |
| Caesarean section >1, no. (%) | | | 6/40 (15.00) | | 5/58 (8.62) | | n.s. | | |  |  |
| 1 | | | 4/40 (10.00) | | 2/58 (3.45) | |  | | |  |  |
| 2 | | | 2/40 (5.00) | | 1/58 (1.72) | |  | | |  |  |
| 3 | | | 0/40 (0.00) | | 2/58 (3.45) | |  | | |  |  |
|  | | |  | |  | |  | | |  |  |
| Vaginal delivery >1, no. (%) | | | 7/40 (17.50) | | 28/58 (48.28) | | 0.003 | | |  |  |
| 1 | | | 4/40 (10.00) | | 13/58 (22.41) | |  | | |  |  |
| 2 | | | 3/40 (7.50) | | 10/58 (17.24) | |  | | |  |  |
| 3 | | | 0/40 (0.00) | | 5/58 (8.62) | |  | | |  |  |
|  | | |  | |  | |  | | |  |  |
| Smoking history, no. (%) | | | 8/40 (20.00) | | 5/58 (8.62) | | n.s. | | |  |  |
| *Statistics, Fisher`s exact test  n.s., not significant | | |  | |  | |  | | |  |  |
| **Table S11. Relationship between patient characteristics and *PIK3CA* mutation status in normal endometrium related to Table 1.** | | | | | | | | | | |  |
|  | | Value (%) | | | | | | *Statistics | | |  |
|  | | *PIK3CA* wild type | | *PIK3CA* mutated | | | |  |  |  |  |
| Characteristic | | (N=79) | | (N=19) | | | |  |  |  |  |
| Gravidity >1, no. (%) | | 42/79 (53.16) | | 8/19 (42.11) | | | | n.s. | | |  |
| 1 | | 18/79 (22.78) | | 3/19 (15.79) | | | |  | | |  |
| 2 | | 14/79 (17.72) | | 2/19 (10.53) | | | |  | | |  |
| 3 | | 6/79 (7.59) | | 3/19 (15.79) | | | |  | | |  |
| 4 | | 3/79 (3.80) | | 0/19 (0.00) | | | |  | | |  |
| 6 | | 1/79 (1.27) | | 0/19 (0.00) | | | |  | | |  |
|  | |  | |  | | | |  | | |  |
| Parity >1, no. (%) | | 37/79 (46.84) | | 7/19 (36.84) | | | | n.s. | | |  |
| 1 | | 16/79 (20.25) | | 4/19 (21.05) | | | |  | | |  |
| 2 | | 14/79 (17.72) | | 1/19 (5.26) | | | |  | | |  |
| 3 | | 7/79 (8.86) | | 2/19 (10.53) | | | |  | | |  |
|  | |  | |  | | | |  | | |  |
| Abortion or stillborn >1, no. (%) | | 11/79 (13.92) | | 3/19 (15.79) | | | | n.s. | | |  |
| 1 | | 6/79 (7.59) | | 3/19 (15.79) | | | |  | | |  |
| 2 | | 4/79 (5,06) | | 0/19 (0.00) | | | |  | | |  |
| 3 | | 1/79 (1.27) | | 0/19 (0.00) | | | |  | | |  |
|  | |  | |  | | | |  | | |  |
| Caesarean section >1, no. (%) | | 9/79 (11.39) | | 2/19 (10.53) | | | | n.s. | | |  |
| 1 | | 5/79 (6.33) | | 1/19 (5.26) | | | |  | | |  |
| 2 | | 3/79 (3.80) | | 0/19 (0.00) | | | |  | | |  |
| 3 | | 1/79 (1.27) | | 1/19 (5.26) | | | |  | | |  |
|  | |  | |  | | | |  | | |  |
| Vaginal delivery >1, no. (%) | | 29/79 (36.71) | | 6/19 (31.58) | | | | n.s. | | |  |
| 1 | | 13/79 (16.46) | | 4/19 (21.05) | | | |  | | |  |
| 2 | | 11/79 (13.92) | | 2/19 (10.53) | | | |  | | |  |
| 3 | | 5/79 (6.33) | | 0/19 (0.00) | | | |  | | |  |
|  | |  | |  | | | |  | | |  |
| Smoking history, no. (%) | | 12/79 (15.19) | | 1/19 (5.26) | | | | n.s. | | |  |
| *Statistics, Fisher`s exact test  n.s., not significant | |  | |  | | | |  | | |  |
| **Table S12. Relationship between patient characteristics and *PPP2R1A* mutation status in normal endometrium related to Table 1.** | | | | | | | | | | | |
|  | Value (%) | | | | | | | | *Statistics | | |
|  | *PPP2R1A* wild type | | | | | *PPP2R1A* mutated | | |  |  |  |
| Characteristic | (N=83) | | | | | (N=15) | | |  |  |  |
| Gravidity >1, no. (%) | 44/83 (53.01) | | | | | 6/15 (40.00) | | | n.s. | | |
| 1 | 18/83 (21.69) | | | | | 3/15 (20.00) | | |  | | |
| 2 | 14/83 (16.87) | | | | | 2/15 (13.33) | | |  | | |
| 3 | 8/83 (9.64) | | | | | 1/15 (6.67) | | |  | | |
| 4 | 3/83 (3.61) | | | | | 0/15 (0.00) | | |  | | |
| 6 | 1/83 (1.20) | | | | | 0/15 (0.00) | | |  | | |
|  |  | | | | |  | | |  | | |
| Parity >1, no. (%) | 38/83 (45.78) | | | | | 6/15 (40.00) | | | n.s. | | |
| 1 | 16/83 (19.28) | | | | | 4/15 (26.67) | | |  | | |
| 2 | 13/83 (15.66) | | | | | 2/15 (13.33) | | |  | | |
| 3 | 9/83 (10.84) | | | | | 0/15 (0.00) | | |  | | |
|  |  | | | | |  | | |  | | |
| Abortion or stillborn >1, no. (%) | 13/83 (15.66) | | | | | 1/15 (6.67) | | | n.s. | | |
| 1 | 8/83 (9.64) | | | | | 1/15 (6.67) | | |  | | |
| 2 | 4/83 (4.82) | | | | | 0/15 (0.00) | | |  | | |
| 3 | 1/83 (1.20) | | | | | 0/15 (0.00) | | |  | | |
|  |  | | | | |  | | |  | | |
| Caesarean section >1, no. (%) | 9/83 (10.84) | | | | | 2/15 (13.33) | | | n.s. | | |
| 1 | 4/83 (4.82) | | | | | 2/15 (13.33) | | |  | | |
| 2 | 3/83 (3.61) | | | | | 0/15 (0.00) | | |  | | |
| 3 | 2/83 (2.41) | | | | | 0/15 (0.00) | | |  | | |
|  |  | | | | |  | | |  | | |
| Vaginal delivery >1, no. (%) | 30/83 (36.14) | | | | | 5/15 (33.33) | | | n.s. | | |
| 1 | 13/83 (15.66) | | | | | 4/15 (26.67) | | |  | | |
| 2 | 12/83 (14.46) | | | | | 1/15 (6.67) | | |  | | |
| 3 | 5/83 (6.02) | | | | | 0/15 (0.00) | | |  | | |
|  |  | | | | |  | | |  | | |
| Smoking history, no. (%) | 11/83 (13.25) | | | | | 2/15 (13.33) | | | n.s. | | |
| *Statistics, Fisher`s exact test  n.s., not significant |  | | | | |  | | |  | | |
